# Effectiveness and Safety of Preoperative Nutritional Interventions on Surgical Outcomes in Patients Undergoing Metabolic and Bariatric Surgery: A Systematic Review and Meta-Analysis

**DOI:** 10.3390/nu17091533

**Published:** 2025-04-30

**Authors:** Daniel Simancas-Racines, Claudia Reytor-González, Juan Marcos Parise-Vasco, Jaime Angamarca-Iguago, Eloisa Garcia-Velasquez, Ashley Carolina Cuzco-Macias, Evelyn Frias-Toral, Luigi Schiavo

**Affiliations:** 1 Universidad UTE, Facultad de Ciencias de la Salud Eugenio Espejo, Centro de Investigación en Salud Pública y Epidemiología Clínica (CISPEC), Quito 170527, Ecuador; dsimancas@ute.edu.ec (D.S.-R.); claudia.reytor@ute.edu.ec (C.R.-G.); jaime.angamarca@ute.edu.ec (J.A.-I.); 2 Clinical Nutrition Service, Grupo Hospitalario Kennedy, Guayaquil 090902, Ecuador; eloisa.garciavelasquez@gmail.com; 3 Servicio de Cardiología, Hospital General de Agudos Dr. Juan A. Fernández, Buenos Aires C1425AGP, Argentina; carolinacuzcomacias@gmail.com; 4 Universidad UTE, Posgrados de Ciencias de la Salud, Maestría de Epidemiología con Mención en Investigación Clínica Aplicada, Quito 170527, Ecuador; 5 Universidad Espíritu Santo, Escuela de Medicina, Samborondón 0901952, Ecuador; evelynft@gmail.com; 6 Division of Research, Texas State University, 601 University Dr., San Marcos, TX 78666, USA; 7 Department of Medicine, Surgery and Dentistry “Scuola Medica Salernitana”, University of Salerno, 84081 Baronissi, Italy; 8 National Biodiversity Future Center (NBFC), 90133 Palermo, Italy

**Keywords:** very low-calorie diet, low-calorie diet, bariatric surgery, preoperative nutrition, perioperative outcomes, meta-analysis

## Abstract

**Background**: Preoperative nutritional interventions, including low-calorie diets (LCDs) and very low-calorie diets (VLCDs), are commonly implemented in metabolic and bariatric surgery. This systematic review and meta-analysis aimed to evaluate the efficacy and safety of preoperative dietary interventions in patients undergoing bariatric surgery, with primary outcomes including perioperative complications, operative time, and length of hospital stay. **Methods**: A systematic review and meta-analysis were conducted, including studies that compared LCD and VLCD with regular diets in adults undergoing bariatric surgery. The primary outcomes assessed were perioperative complications, operative time, and length of hospital stay. Random- and fixed effects models were used for quantitative synthesis. Risk of bias was evaluated using the Cochrane Risk of Bias tool and ROBINS-I, while the certainty of evidence was assessed using the GRADE approach. **Results**: Eight trials comprising 1197 patients were included in the meta-analysis. VLCDs were associated with a significant reduction in perioperative complications (OR 0.59; 95% CI: 0.37–0.94; *p* = 0.03), whereas LCDs showed no significant effect on complications (OR 1.64; 95% CI: 0.71–3.78; *p* = 0.25). No significant reduction in operative time was observed (MD −2.64 min; 95% CI: −6.01 to 0.73; *p* = 0.12). Hospital stay was slightly reduced (MD −0.17 days; *p* = 0.0001), though the clinical significance remains uncertain. The certainty of evidence was low, primarily due to the risk of bias and small sample sizes. **Conclusions**: VLCDs may lower the risk of perioperative complications, while LCDs do not appear to provide this benefit. However, the evidence is limited by methodological heterogeneity and low certainty. Further high-quality studies are needed to establish optimal preoperative nutritional protocols.

## 1. Introduction

Obesity is a chronic disease with a high global prevalence, significantly impacting physical, psychological, and economic health. It is estimated to affect over 650 million people worldwide [1,2,3,4,5,6]. It is associated with metabolic conditions such as type 2 diabetes, hypertension, and dyslipidemia, in addition to increasing the risk of cardiovascular diseases and certain cancers [7,8,9,10,11,12]. In patients with severe obesity (body mass index (BMI) ≥ 40 kg/m^2^ or ≥35 kg/m^2^ with comorbidities), traditional strategies, including lifestyle modifications [13,14] and pharmacological therapies [15], often fail to achieve sustained results, highlighting the need for more effective approaches such as bariatric surgery (BS) [16,17,18,19,20,21].

BS is recognized as the most effective treatment for severe obesity, providing significant benefits in weight loss, improvement of metabolic comorbidities, and an enhanced quality of life and longevity [22]. However, its success depends not only on the surgical procedure itself but also on proper preoperative preparation [23]. Preoperative interventions are essential for optimizing the patient’s condition before surgery, aiming to reduce surgical complications and improve metabolic outcomes [24,25]. Among these interventions, non-pharmacological strategies, particularly nutritional approaches, play a crucial role in managing obesity and obesity-related disorders both before and after surgery [26,27].

Low-calorie diets (LCD) and very-low-calorie diets (VLCD) are two established nutritional strategies widely used in obesity management [28]. LCDs typically provide 800–1200 kcal/day, while VLCDs supply less than 800 kcal/day, often with a high-protein, nutrient-enriched formulation to preserve lean mass. Within these categories, low-calorie ketogenic diets (LCKD) and very-low-calorie ketogenic diets (VLCKD) represent specific ketogenic adaptations characterized by severe carbohydrate restriction and nutritional ketosis induction, aiming to enhance fat loss and metabolic control [29,30]. Recently, the term very-low-energy ketogenic therapy (VLEKT) was introduced to emphasize the therapeutic nature of VLCKDs, particularly in clinical settings [31].

Ketogenic diets, such as LCKD and VLCKDs, have demonstrated efficacy in promoting preoperative weight loss, decreasing waist circumference, and facilitating bariatric surgery. In addition, VLCKDs have been associated with reductions in liver volume, which is a crucial factor for minimizing surgical complexity [32,33]. Moreover, these dietary strategies can lower the likelihood of requiring open surgery and reduce perioperative complications, positioning them as a safe and effective preoperative nutritional approach for patients with obesity [34].

Several studies by Schiavo et al. have consistently shown that VLCKDs represent a valuable strategy in the preoperative management of patients undergoing bariatric surgery. These protocols, often formulated with micronutrient enrichment and severe carbohydrate restriction, have been associated with significant reductions in both body weight and liver volume—two key parameters that directly contribute to decreasing surgical complexity and improving intraoperative safety, particularly during laparoscopic procedures [35]. Furthermore, randomized trials comparing VLCKDs to standard low-calorie diets have shown that patients following the ketogenic approach demonstrate a superior preservation of fat-free mass and resting metabolic rate after interventions such as intragastric balloon placement, suggesting a metabolic advantage that may benefit long-term outcomes [36]. Complementary findings by Barrea et al. further support the use of the VLCKD in this context, highlighting its role in effectively reducing hepatic and visceral fat while also enhancing postoperative metabolic recovery [33].

Although these preoperative dietary strategies have shown potential to induce short-term weight loss and metabolic improvements, their impact on clinically relevant surgical outcomes remains unclear. Given the increasing implementation of these interventions prior to metabolic and bariatric surgery, it is essential to understand their effectiveness and safety not only in terms of anthropometric changes but also regarding perioperative complications, operative time, and hospital length of stay. Therefore, this systematic review aims to synthesize the current evidence on the role of preoperative nutritional interventions in optimizing surgical outcomes and improving overall perioperative management in patients undergoing bariatric procedures.

## 2. Methods

A systematic review was conducted to assess the effectiveness and safety of preoperative dietary interventions on surgical outcomes in patients undergoing metabolic and bariatric surgery (MBS). The methodology followed the recommendations of the Cochrane Handbook for Systematic Reviews of Interventions [37], and reporting followed the Preferred Reporting Items for Systematic Reviews and Meta-Analysis (PRISMA) guidelines [38]. The review protocol was prospectively registered in PROSPERO (CRD42024622203). The research question was structured according to the PICO framework: Population (P): Adult patients undergoing metabolic and bariatric surgery, Intervention (I): Low and very low-calorie preoperative diets, Comparison (C): usual diet or any other dietary intervention. Outcome (O): Operative time, length of hospital stay, perioperative complications, and changes in weight or BMI.

### 2.1. Search Strategy

A comprehensive literature search was conducted on six electronic databases: MEDLINE/PubMed, Cochrane Central Register of Controlled Trials, LILACS, BVS, Scopus, and Epistemonikos. The search included trials published in English and Spanish up to November 2024. The strategy combined terms related to bariatric surgery, metabolic surgery, specific dietary interventions, and the preoperative period. The detailed search strategies are provided in Table S1.

In addition, we searched clinical trials registries, including ClinicalTrials.gov and the World Health Organization International Clinical Trials Registry Platform (WHO ICTRP). Grey literature was identified by searching Google Scholar. Reference lists of included studies and relevant systematic reviews were manually reviewed to identify additional eligible studies.

### 2.2. Eligibility Criteria

We included randomized controlled trials, non-randomized controlled clinical trials, and cohort studies with control groups that evaluated preoperative dietary interventions in patients undergoing metabolic and bariatric surgery. Studies had to include adult patients and report at least one of the following outcomes: preoperative weight loss, operative time, hospital stay or perioperative complications. We excluded systematic reviews, case reports, letters to the editor, studies without a comparison group, studies that did not specifically analyze preoperative dietary interventions, and studies that focused only on other preoperative interventions, such as pharmacological treatments or intragastric balloons.

### 2.3. Study Selection

Two independent reviewers (JMPV and ACCM) conducted the study selection process using the Rayyan web-based tool (https://rayyan.qcri.org). The selection process was carried out in two stages: first, the titles and abstracts of all references identified in the initial search were screened to assess their potential eligibility. The full texts of the pre-selected studies were retrieved and reviewed to confirm compliance with the predefined inclusion criteria. Disagreements between reviewers at any stage of the selection process were resolved by consensus, with a third reviewer (DSR) acting as an arbitrator when necessary.

### 2.4. Data Extraction

Data extraction was conducted using a standardized matrix by four reviewers (JMPV, ACCM, CRG, JAI), who worked collaboratively to reach consensus on the extracted data elements. The following information as extracted from each study: title; study characteristics; patient demographics; details of the dietary interventions, including diet type, duration, calorie intensity, dietary composition, surgical procedure performed; and outcome measures such as preoperative weight loss, operative time, hospital stay, complications, and the definition and classification of low-calorie diet (LCD), very low-calorie diet (VLCD), and other types of diet used in each study. Any disagreements during data extraction were resolved by consensus with a fourth reviewer (DSR).

### 2.5. Risk Bias Assessment of Included Studies

The quality of the included studies was assessed independently by two reviewers (JMPV, ACCM) using the Cochrane Risk of Bias tool for randomized controlled trials [37] and the ROBINS-I tool for non-randomized trials [39]. Disagreements in the quality assessment were resolved by consensus or arbitration by a third reviewer (DSR).

### 2.6. Data Synthesis and Statistical Analysis

A qualitative synthesis of study characteristics, interventions, and outcomes was conducted. For quantitative meta-analysis, trials comparing low-calorie diets (including LCD and VLCD) with usual diet were pooled, regardless of the specific duration and caloric intensity of the dietary intervention. This approach was chosen because of the heterogeneity in the definitions and protocols of the dietary interventions in the included studies.

The choice between fixed effects and random effects models was based on an assessment of clinical and statistical heterogeneity. For the primary analysis, a random effects model was used due to the diverse nature of the included studies. Statistical heterogeneity was evaluated using the *I*^2^ statistic. Substantial heterogeneity was defined as *I*^2^ > 50%, in which case the results from the random effects model were prioritized. For outcomes with low to moderate heterogeneity (*I*^2^ ≤ 50%), sensitivity analyses were conducted to compare the results of both models [37].

For dichotomous outcomes (operative complications), results were reported as odds ratios (OR) with corresponding 95% confidence intervals (CI). Continuous outcomes (preoperative weight loss, operative time, and hospital stay) were analyzed using mean differences (MD), with 95% CIs reported for all effect estimates. For each outcome, the specific weight of each study and its contribution to the overall estimated effect was assessed. Data were analyzed using Review Manager (RevMan) v5.3 software. The certainty of evidence for each outcome was assessed using the Grading of Recommendations Assessment, Development and Evaluation (GRADE) approach using GRADEpro software [40].

Publication bias was planned to be assessed using funnel plots and Egger’s test when 10 or more studies could be pooled for an outcome [37]. However, as fewer than 10 trials were available for meta-analysis of each outcome, a formal assessment of publication bias was not conducted.

## 3. Results

**Figure 1:**
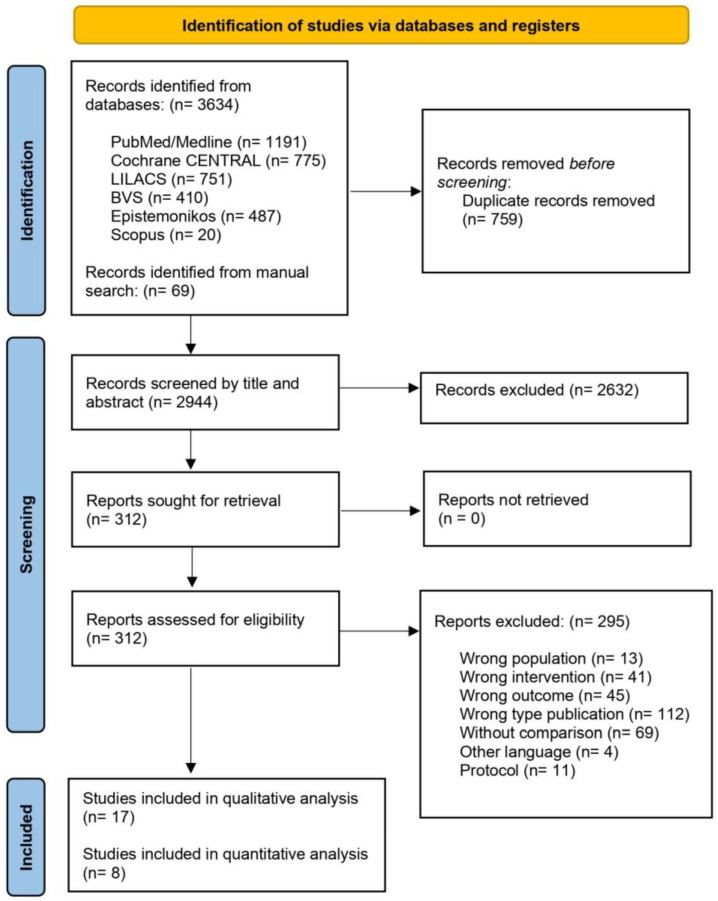
PRISMA flowchart of systematic review selection process and included studies.

The systematic search yielded a total of 3703 records, including 3634 records identified through electronic database searches and 69 records identified through manual searches. After removing 759 duplicates, 2944 records were screened by title and abstract, excluding 2632. After the initial screening, 312 full-text articles were assessed for eligibility. Of these, 295 reports were excluded for the following reasons: wrong population (n = 13), wrong intervention (n = 41), wrong outcome (n = 45), wrong publication type (n = 112), without comparison group (n = 69), non-eligible language (n = 4), or being protocol papers (n = 11) (Table S2). The final analysis included 17 studies for qualitative synthesis [30,41,42,43,44,45,46,47,48,49,50,51,52,53,54,55,56] and 8 studies with data for quantitative meta-analysis [41,46,47,50,51,52,54,56] (Figure 1).

### 3.1. Characteristics of Studies

The 17 included studies had a variety of methodological designs, ranging from randomized controlled trials [48,49,53,54] to retrospective cohort studies [30,41] and nonrandomized prospective designs [47,52]. Geographically, the studies were conducted mainly in Europe (Italy, The Netherlands, Spain, Sweden, Turkey, United Kingdom), with additional studies from Brazil, Egypt, Thailand, and the United States.

The most commonly performed surgical procedures were laparoscopic Roux-en-Y gastric bypass (LRYGB) [45,48,50,51,54,56] and laparoscopic sleeve gastrectomy (LSG) [30,41,47,49], with some trials including both procedures [46,53]. Most of the patients had obesity-related comorbidities, mainly type 2 diabetes, hypertension, dyslipidemia, and obstructive sleep apnea.

Preoperative dietary interventions varied in duration (from 10 days to 4 weeks) and caloric intensity. Very low-calorie diets (VLCD/VLCKD) typically provided 600–800 kcal/day [30,46,47,48,49,51,52,55,56], while low-calorie diets (LCD) ranged from 800 to 1600 kcal/day [52,53]. Some trials used commercial formulations such as Optifast [51,53] or Prodimed [50], formulations enriched with omega-3 fatty acids [48,54], or immunonutrition [49]. Controls ranged from standard care without specific dietary intervention [46,47,51,52] to active comparators, including standard LCDs [30,44,53] or the Mediterranean diet [43] (Table 1).

### 3.2. Qualitative Synthesis of Key Outcomes

The evidence from the 17 included studies was synthesized to describe the impact of preoperative nutritional interventions on major surgical and postoperative outcomes in patients undergoing metabolic and bariatric surgery. Findings were categorized by outcome type, with additional analyses of variations in operative parameters, liver volume reduction, perioperative complications, and postoperative recovery.

#### 3.2.1. Anthropometric Changes (Weight, BMI)

All studies reported significant weight loss with preoperative dietary interventions. The magnitude of weight loss varied by intervention type, duration, and baseline BMI. VLCD/VLCKD interventions consistently produced greater weight loss compared to standard diets or less restrictive interventions.

Albanese et al. reported significantly greater weight loss with VLCKD (5.8 ± 2.4 kg) compared to VLCD (4.8 ± 2.5 kg, *p* = 0.008) [30]. Similarly, Contreras et al. [53] found that VLCD resulted in greater weight loss (5.8%) compared to LCD (4.2%, *p* = 0.004). Erdem et al. [43] reported superior results with VLCKD-SDM compared to the Mediterranean diet (weight loss 2.7 kg/m^2^ vs. 1.4 kg/m^2^, *p* < 0.05).

Intervention duration positively correlated with weight loss. In Yolsuriyanwong et al. [55], patients with BMI ≥ 50 kg/m^2^ who followed a 2-week VLCD lost significantly more weight than those with BMI < 50 kg/m^2^ who followed a 1-week VLCD (7.0 ± 2.3 kg vs. 4.4 ± 1.8 kg, *p* < 0.001). Nieuwenhove et al. [51] demonstrated that a 14-day VLCD resulted in a mean weight loss of 4.9 kg compared to 0.4 kg in the control group (*p* < 0.001).

Schouten et al. [50] found no significant differences in weight loss between a commercially formulated VLCD (Prodimed, 5.9 kg) and a standard low-carbohydrate diet (6.0 kg), suggesting that caloric restriction rather than specific formulation may be the key factor.

The addition of specific nutrients showed variable effects. Ruiz-Tovar et al. [49] reported greater weight loss with an immunonutrition formula (15.3%) compared to a high-protein formula (12.3%) or a regular diet (7.7%, *p* = 0.014). Similarly, Ruiz-Tovar et al. [48] found greater weight loss with an omega-3-enriched formula (14.1 ± 5.8%) compared to a standard formula (10.6 ± 7.7%, *p* = 0.024).

#### 3.2.2. Operative Parameters

Findings regarding operative time were inconsistent. Several studies reported no significant differences [48,49,50,51,52,53], while Khemtong et al. found significantly shorter operative times following VLCD [46]. Several studies documented subjective improvements in operative difficulty, particularly in relation to liver exposure and retraction [46,47,52,56].

#### 3.2.3. Changes in Liver Volume

A consistent reduction in liver volume has been observed following nutritional interventions. Edholm et al. reported a 12% reduction (*p* < 0.001) after 4 weeks with LCD [52], while Chakravartty et al. observed a 23% reduction with VLCD compared to only 2% in the control group (*p* = 0.03) [56].

#### 3.2.4. Perioperative Complications

The effect on perioperative complications varied between studies. Nieuwenhove et al. reported significantly fewer postoperative complications in the VLCD group compared with controls (8 vs. 18, *p* = 0.04) [51], while other studies found no significant differences in complication rates [47,48,49,53,57].

#### 3.2.5. Postoperative Outcomes

Length of hospital stay showed mixed results. Albanese et al. reported a shorter length of stay for the VLCKD group compared with the VLCD group [30], while other studies found no significant differences [50,52,53]. Some studies reported improvements in inflammatory markers, particularly lower C-reactive protein levels [48,49], and sustained benefits in glycemic control in diabetic patients [45,46].

#### 3.2.6. Additional Relevant Findings

Acceptance of and adherence to dietary interventions also varied. Schouten et al. found that aspects such as “taste”, “variety” and “satiety” were significantly better in the standard diet group than in the commercial VLCD group [50], suggesting that patient preference should be considered when selecting preoperative dietary interventions.

### 3.3. Risk of Bias Assessment of Included Studies

#### 3.3.1. Risk of Bias in Included Randomized Controlled Trials

The assessment of the risk of bias in randomized controlled trials showed that the methodological quality varied between the different areas. Most studies showed a low risk of bias in random sequence generation, with ten studies explicitly reporting computer-generated randomization or random number tables [42,44,45,48,49,50,51,53,54,56]. Allocation concealment was adequately described in seven studies using sealed envelope systems [40,43,48,49,51,52,57], while three studies provided insufficient details [44,48,49]. Blinding of participants and personnel presented the greatest challenge, with eight studies showing a high risk of performance bias [42,44,45,50,51,53,54,56] due to participants’ awareness of dietary assignments; only two studies achieved low risk through double-blind designs with similar nutritional supplements [48,49]. For outcome assessment blinding, seven studies demonstrated low risk by explicitly blinding outcome assessors [48,49,50,51,53,54,56], two had mixed risk assessments [42,54], and one received high-risk rating for lack of information [43]. Most studies showed a low risk of attrition bias, with only one study rated high-risk due to differential attrition between groups [44]. Regarding other biases, seven studies showed low risk [30,42,44,50,51,53,56], while three had unclear risk due to issues such as small sample size, gender imbalance, or per-protocol analyses [45,48,49] (Figures S1 and S2).

#### 3.3.2. Risk of Bias in Included Non-Randomized Studies

The risk of bias for the seven non-randomized trials was assessed using the ROBINS-I tool [39]. Three studies were identified as having a serious risk of bias [30,41,46], while four were identified as having a moderate risk [43,47,52,55]. Confounding was identified as the most problematic area, with all studies having a moderate to serious risk due to non-randomized group allocation, self-selection into groups, and significant baseline differences. Selection bias was generally moderate, while the classification of interventions was consistently rated as low risk. Deviations from the intended interventions, missing data and outcome measures were predominantly of low to moderate risk, with notable concerns such as higher dropout rates in more restrictive diet groups [43] (Figures S3 and S4).

### 3.4. Quantitative Meta-Analysis

This systematic review identified eight studies that evaluated the preoperative effects of LCD and VLCD compared to regular diets in patients undergoing metabolic and bariatric surgery. A total of 1197 patients were included for the analysis of complications, 748 patients for operative time and 636 patients for hospital length of stay.

#### 3.4.1. Operative Complications of Any Type

The primary analysis using a random effects model showed that low-calorie (LCD) and very low-calorie (VLCD) diets were not significantly associated with a reduction in the risk of complications compared with usual diets (OR 0.78; 95% CI: 0.49–1.26; *p* = 0.31), with low heterogeneity between studies (*I*^2^ = 14%). Subgroup analysis revealed significant differences between dietary intervention types (*p* = 0.04; *I*^2^ = 77.2%).

**Figure 2:**
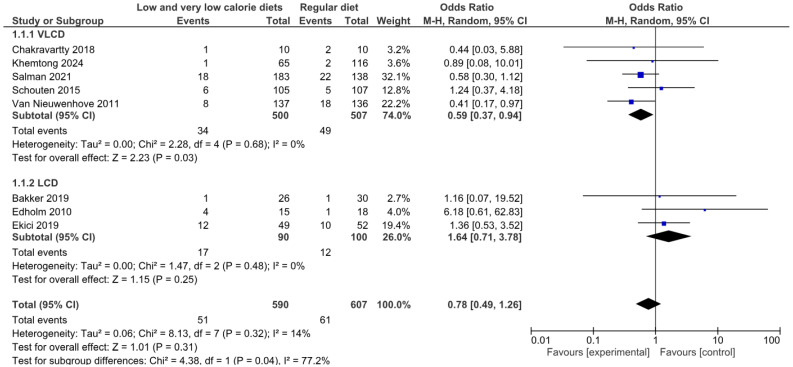
Meta-analysis of operative complications of any type [41,46,47,50,52,54,56].

VLCDs showed a significant reduction in the risk of complications (OR 0.59; 95% CI: 0.37–0.94; *p* = 0.03; *I*^2^ = 0%), based on five studies and 1007 patients. Conversely, LCDs showed a non-significant trend towards a possible increase in complications (OR 1.64; 95% CI: 0.71–3.78; *p* = 0.25; *I*^2^ = 0%), based on three studies with 190 patients (Figure 2).

Sensitivity analysis using a fixed effects model showed similar results (overall OR 0.76; 95% CI: 0.51–1.13; *p* = 0.18), with consistent estimates for VLCD (OR 0.58; 95% CI: 0.37–0.93; *p* = 0.02) and LCD (OR 1.70; 95% CI: 0.75–3.86; *p* = 0.20) (Figure 2).

According to the GRADE approach, the certainty of the evidence for this outcome was rated as very low, mainly due to the methodological limitations inherent in observational studies, risk of bias, and the imprecision of the global effect estimates (Table S3).

#### 3.4.2. Operative Time

The primary analysis, using a random effects model of five studies, showed that low-calorie (LCD) and very low-calorie (VLCD) diets did not significantly reduce operative time compared with a usual diet (MD −2.64 min; 95% CI: −6.01 to 0.73; *p* = 0.12), with moderate to high heterogeneity between studies (*I*^2^ = 63%). Subgroup analysis showed significant differences between dietary intervention types (*p* = 0.002; *I*^2^ = 89.9%). LCDs showed a significant reduction in operative time (MD −5.38 min; 95% CI: −7.59 to −3.16; *p* < 0.00001; *I*^2^ = 0%) in two studies with 134 patients. In contrast, VLCDs showed no significant effect on this parameter (MD −0.57 min; 95% CI: −2.58 to 1.45; *p* = 0.58; *I*^2^ = 0%) in three trials with 614 patients.

**Figure 3:**
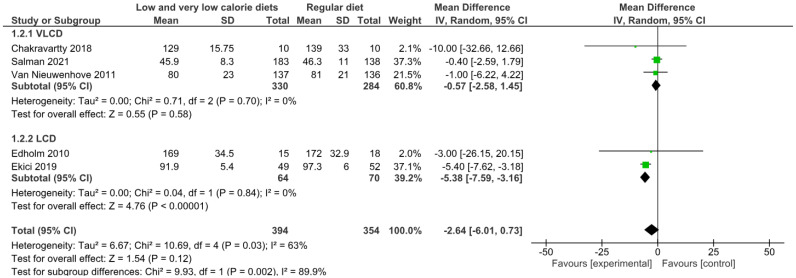
Meta-analysis of operative time [41,47,51,52,56].

Sensitivity analysis using a fixed effects model showed a statistically significant overall effect (MD −2.75 min; 95% CI: −4.24 to −1.26; *p* = 0.0003), while subgroup results remained virtually unchanged. This discrepancy between models suggests that the conclusion regarding an overall reduction in operating time is sensitive to the statistical model chosen (Figure 3).

According to the GRADE approach, the certainty of this evidence was rated as very low because of the limitations of the observational studies, the moderate to high heterogeneity observed (*I*^2^ = 63%), and the sensitivity of the results to the statistical model used, which leads to uncertainty about the magnitude and statistical significance of the overall effect (Table S3).

#### 3.4.3. Length of Hospital Stay

**Figure 4:**
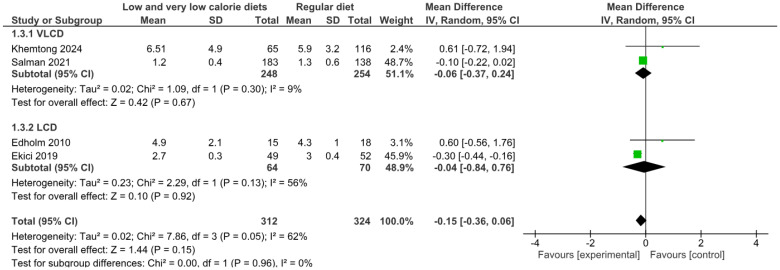
Meta-analysis of length of hospital stay [41,46,47,52].

The primary analysis, using a random effects model of four trials, showed that low-calorie (LCD) and very low-calorie (VLCD) diets did not significantly reduce hospital length of stay compared with usual diets (MD −0.15 days; 95% CI: −0.36 to 0.06; *p* = 0.15), with moderate to high heterogeneity between trials (*I*^2^ = 62%). Notably, subgroup analysis showed no significant differences between dietary intervention types (*p* = 0.96; *I*^2^ = 0%). (Figure 4).

VLCDs did not significantly affect the length of hospital stay (MD −0.06 days; 95% CI: −0.37 to 0.24; *p* = 0.67; *I*^2^ = 9%) in two trials with 502 patients. Similarly, LCDs did not show a significant effect (MD −0.04 days; 95% CI: −0.84 to 0.76; *p* = 0.92; *I*^2^ = 56%) in two studies with 134 patients.

Sensitivity analysis using a fixed effects model yielded substantially different results, showing a statistically significant overall effect (MD −0.17 days; 95% CI: −0.26 to −0.09; *p* = 0.0001) and significant differences between subgroups (*p* = 0.03; *I*^2^ = 77.7%). This analysis showed that LCDs significantly reduced hospital length of stay (MD −0.29 days; 95% CI: −0.42 to −0.15; *p* < 0.0001), whereas VLCDs showed no significant effect (MD −0.09 days; 95% CI: −0.21 to 0.02; *p* = 0.11). This large discrepancy between models suggests that conclusions about length of stay are highly sensitive to the statistical model used.

According to the GRADE approach, the certainty of the evidence for this outcome was rated as very low. This assessment is justified by the methodological limitations of the included observational studies, the substantial heterogeneity (*I*^2^ = 62%), and, in particular, the marked discrepancy between the results obtained with different statistical models, which indicates a significant instability in the estimates and significantly reduces the confidence in any conclusions regarding this outcome (Table S3).

## 4. Discussion

This systematic review examined the efficacy and safety of preoperative nutritional interventions on surgical outcomes in patients undergoing metabolic and bariatric surgery. The primary analysis, using a random effects model, found that VLCD may significantly reduce the risk of postoperative complications, whereas low-calorie diets (LCD) showed a non-significant trend toward a possible increase in complications. This differential effect may support the importance of distinguishing between types of dietary intervention, rather than treating all approaches to caloric restriction as equivalent. However, there were differences in complication rates between the included trials. While Ruiz-Tovar et al. and Schouten et al. found no significant differences [48,49,50], Albanese et al. [30] and Khemtong et al. [46] reported fewer adverse events with VLCD. This variability may also be due to differences in surgical procedures, such as sleeve gastrectomy versus gastric bypass.

Complication rates varied among the included trials. While Ruiz-Tovar et al. [48,49] and Schouten et al. [50] found no significant differences, Albanese et al. [30] and Khemtong et al. [46] reported fewer adverse events with VLCD. This variability may also be attributed to differences in surgical procedures, such as sleeve gastrectomy versus gastric bypass. Regarding surgical parameters, our random effects model analysis did not find a statistically significant reduction in overall operative time. However, subgroup analysis revealed that LCD, but not VLCD, significantly reduced operative time. This discrepancy suggests that caloric restriction intensity does not necessarily predict surgical efficiency. Additionally, our primary analysis did not find a significant reduction in hospital length of stay, although sensitivity analysis using a fixed effects model showed a statistically significant but clinically irrelevant reduction.

An important physiological effect observed in several trials was the significant reduction in liver volume following preoperative dietary interventions. Studies by Edholm et al. [52], Chakravartty et al. [56], and Salman et al. [47] consistently reported a reduction in liver volume ranging from 12% to 23%. This reduction is likely to facilitate the technical aspects of bariatric surgery, in particular by improving access to the gastroesophageal junction and reducing the need for aggressive liver retraction, which may explain the improvement in surgical outcomes despite modest reductions in operative time. Similarly, anthropometric results showed consistent weight loss across all dietary intervention studies, with VLCD/VLCKD interventions potentially resulting in greater weight loss than less restrictive approaches. The magnitude of preoperative weight loss correlated with the duration and intensity of the intervention, with studies reporting reductions ranging from 4.4 kg to 15.3% of baseline weight.

The findings of this review align with previous systematic reviews highlighting the clinical benefits of very low-calorie diets in metabolic and bariatric surgery. Consistent with Stenberg et al. [58], our analysis suggests that VLCDs may reduce operative complications. Regarding operative difficulty, our findings generally agree with other reviews indicating that preoperative dietary interventions, particularly VLCD/VLCKD, may reduce perceived operative difficulty. This observation is consistent with Stenberg et al., who reported that two studies documented a reduction in surgical difficulty following VLCD interventions despite inconsistent effects on operative time [58]. Holderbaum et al. [29] also recognized the potential of preoperative diets to reduce surgical difficulty by reducing liver volume and intra-abdominal fat.

The results on operative time and hospital length of stay, based on the random effects model, differ from some previous findings. While Stenberg et al. [58] observed a reduction in operative time primarily for upper gastrointestinal procedures, our primary analysis did not find a significant effect on overall operative time. This discrepancy also contrasts with the findings of Holderbaum et al., who reported inconsistent effects on operative time. These differences likely reflect methodological variations in study selection, outcome definitions, and statistical models used [29]. On the other hand, the evidence on the length of hospital stay was also mixed in the systematic reviews. The meta-analysis found a no statistically significant reduction in length of stay. This finding contrasts with Holderbaum et al. [29] and McKechnie et al. [59], who found no consistent effects on the length of stay. This discrepancy may be due to differences in the primary studies included and our analysis being stratified by diet type. In addition, other factors could explain the observed discrepancies, including differences in inclusion criteria that affect the set of primary studies analyzed. For example, McKechnie et al. restricted their analysis to randomized controlled trials comparing liquid VLCDs with non-VLCD controls [59], whereas our review included a wider range of dietary interventions and study designs. Furthermore, Stenberg et al. [58] evaluated preoperative weight loss interventions across multiple surgical specialties, not just bariatric surgery.

The evidence synthesized in this review has several limitations. The methodological quality of the included studies varied considerably. The risk of bias assessment showed that most randomized trials were at a high or unclear risk of performance bias due to the challenges of blinding participants to dietary interventions. Among non-randomized studies, confounding was the most problematic area, with all studies having a moderate or serious risk of confounding. The heterogeneity of intervention protocols is another major limitation.

The studies used different dietary approaches with different caloric restrictions, intervention periods ranging from 10 days to 4 weeks, and different prescribed dietary compositions. This heterogeneity makes direct comparisons difficult and limits the specificity of recommendations for clinical practice. In addition, most of the trials included had relatively small sample sizes, especially for subgroup analyses. Several studies included fewer than 50 participants, which limits the statistical power to detect clinically significant differences, especially for rare complications. The short follow-up periods in most of the studies, which were limited to immediate postoperative outcomes, prevent evaluation of the long-term effects of preoperative dietary interventions on sustained weight loss or metabolic improvements.

Despite these limitations, this systematic review has several strengths. Our comprehensive search strategy included multiple electronic databases, clinical trial registries, and grey literature, minimizing the risk of missing relevant studies. The inclusion of both randomized and non-randomized trials provided a more comprehensive picture of the available evidence, balancing internal validity with real-world applicability. The assessment of risk of bias used appropriate tools for randomized and non-randomized studies, providing an important context for interpreting the quality of the evidence. However, large, well-designed, randomized controlled trials comparing specific dietary interventions such as VLCD, VLCKD, LCD, and immunonutrition with normal diets, with standardized outcome definitions, are needed to strengthen the evidence base, identify optimal protocols, and investigate the dose–response relationship between intervention intensity, including level and duration of caloric restriction, and surgical outcomes, in order to identify minimally effective interventions that balance benefits with patient acceptability.

In addition, these trials should use rigorous methodology, including allocation concealment, blinded outcome assessment, and intention-to-treat analysis, to address the methodological limitations identified in existing trials. Future trials should also standardize the reporting of perioperative complications using validated classification systems to facilitate comparisons between studies.

## 5. Conclusions

This systematic review and meta-analysis synthesized existing evidence on the effectiveness of preoperative nutritional interventions on surgical outcomes in patients undergoing metabolic and bariatric surgery. Our primary analysis, using random effects models, found that VLCD may reduce the risk of perioperative complications, whereas LCD did not show this benefit. No statistically significant reductions were observed in overall operative time or hospital length of stay, although sensitivity analysis using fixed effects models did show significant but clinically irrelevant reductions in these parameters. It is important to note that the certainty of the evidence, assessed using the GRADE approach, was very low for all primary outcomes, mainly due to the risk of bias in the primary studies, moderate-to-high heterogeneity in some analyses, and imprecision associated with small sample sizes. The substantial heterogeneity in dietary interventions and outcome measures, as well as the sensitivity of findings to the statistical model used, complicates interpretation and generates uncertainty regarding optimal dietary protocols. Additional high-quality studies are required to confirm these findings and specifically evaluate the dose–response relationship between the intensity of caloric restriction, excessive weight loss, other anthropometric measures, and surgical outcomes.

## Figures and Tables

**Table 1:** Characteristics of the included studies.

First Author, Year	Country	Type of Surgery	Sample Size (n)	Mean Age ± SD or Range	Initial BMI ± SD	Intervention Group Description	Control Group Description	Comorbidities Reported
Albanese et al., 2018 [30]	Italy	LSG	178 (72 VLCKD; 106 VLCD)	VLCKD: 43.4 ± 12.1; VLCD: 43.5 ± 11.8	VLCKD: 46.0 ± 6.3; VLCD: 43.6 ± 6.9	3-week VLCKD (700 kcal/day, ≤30 g carbs)	3-week VLCD; ≤800 kcal/day, 80 g carbs	Hypertension (44–50%), T2DM (19–27%), OSA (19–23%), smoking (17–20%)
Bakker et al., 2019 [54]	The Netherlands	LRYGB	62 (LCD: NR; Omega-3: NR)	18–65 years	LCD: 41 ± 6; Omega-3: 43 ± 6	2-week LCD (800 kcal/day)	4-week Omega-3 + normal diet (2000 kcal)	DM (13–27%), dyslipidemia (19–27%), hypertension (37–39%)
Chakravartty et al., 2018 [56]	UK	LRYGB	20 (10 VLCD; 10 control)	NR	Control: 52.75 kg/m^2^; VLCD: 53.4 kg/m^2^	4-week VLCD (800 kcal/day)	Regular diet	Hypertension, asthma, OSA, GERD, PCOS, hypothyroidism
Gils Contreras et al., 2018 [53]	Spain	LRYGB/LSG	86 (43 VLCD; 41 LCD)	18–66 years	47.3 ± 5.2 kg/m^2^	21-day VLCD (800 kcal/day, Optifast^®^)	21-day LCD (1200 kcal/day)	Hypertension, dyslipidemia, OSA, T2DM
Edholm et al., 2011 [52]	Sweden	Lap-GBP	33 (15 LCD; 18 control)	LCD: 34.3 ± 7.53; Control: 42.2 ± 7.05	LCD: 42.9 ± 3.02; Control: 40.8 ± 3.63	4-week LCD (800–1100 kcal/day)	Regular diet	Morbid obesity (BMI > 40)
Ekici et al., 2019 [41]	Turkey	LSG	101 (49 LCD; 52 control)	LCD: 18–59; Control: 18–60	LCD: 45.1 ± 4.4; Control: 44.9 ± 4.1	4-week LCD (1000 kcal/day, high protein)	Regular diet	Hypertension, DM, dyslipidemia, OSA, heart failure
Erdem et al., 2022 [43]	Turkey	NR	30 (15 VLCKD-SDM; 15 MD)	NR	NR	15-day VLCKD-SDM (10–12 kcal/kg/day)	Mediterranean diet (15–20% protein)	NAFLD, OSA, hypertension, T2DM, dyslipidemia
Faria et al., 2014 [42]	Brazil	RYGB	104 (57 liquid VLCD; 47 normal VLCD)	36 ± 10 years	Liquid VLCD: 42.40; Normal VLCD: 39.65	14-day liquid VLCD (760 kcal/day)	14-day normal VLCD (754 kcal/day)	NR
Heinberg et al., 2014 [44]	USA	NR	73 (40 PCD; 33 UDC)	47.33 ± 10.78	49.62 ± 9.52	12-week portion-controlled diet (1300–1600 kcal/day)	Usual dietary care (no caloric target)	NR
Katsogiannos et al., 2019 [45]	Sweden	RYGB	19 (13 RYGB; 6 control)	RYGB: 55 ± 9; Control: 49 ± 5	RYGB: 36.8 ± 4.0; Control: 36.2 ± 4.0	4-week LCD (800–1100 kcal/day)	Routine lifestyle counselling	T2DM
Khemtong et al., 2024 [46]	Thailand	LRYGB/LSG	181 (VLCD: NR; Control: NR)	33.5 ± 10.0 years	60.0 ± 8.5 kg/m^2^	2-week VLCD (800 kcal/day)	Usual diet (no preoperative intervention)	DM, dyslipidemia, NAFLD, hypertension, OSA
Ruiz-Tovar et al., 2019 [48]	Spain	RYGB	40 (20 O3FA; 20 control)	45.9 ± 10 years	41.3 ± 4.2 kg/m^2^	10-day O3FA-enriched formula (900 kcal/day)	High-protein formula (900 kcal/day)	T2DM, hypertension, dyslipidemia, OSA
Ruiz-Tovar et al., 2015 [49]	Spain	LSG	60 (20 IMN; 20 high-protein; 20 control)	43.1 ± 7.2 years	47.8 ± 7.7 kg/m^2^	2-week immunonutrition formula (900 kcal/day)	Regular diet (900 kcal/day)	T2DM, hypertension, dyslipidemia, OSA
Salman et al., 2021 [47]	Egypt	LSG	321 (183 VLCD; 138 control)	18–65 years	VLCD: ~5.8; Control: ~5.5	3-week VLCD (≤800 kcal/day)	No diet	DM (41%), hypertension (41.5%)
Schouten et al., 2015 [50]	The Netherlands	RYGB/Sleeve	212 (105 Prodimed; 107 standard)	Prodimed: 40.2; Standard: 41.7	Prodimed: 42.8; Standard: 43.1	10-day Prodimed VLCD (650 kcal/day)	Standard LCD (647–657 kcal/day)	DM (5–13%), hypertension (37–39%), OSA (9%), joint disease (67%)
Van Nieuwenhove et al., 2011 [51]	Sweden, The Netherlands, Lithuania, Spain, Belgium	RYGB	298 (149 VLCD; 149 control)	VLCD: 39.7; Control: 40.3	VLCD: 43.4; Control: 43.1	14-day VLCD (800 kcal/day, Optifast^®^)	No preoperative diet	T2DM, hypertension, OSA, cardiovascular disease
Yolsuriyanwong et al., 2018 [55]	USA	NR	128 (94 BMI < 50; 34 BMI ≥ 50)	45.6 ± 12.1 years	46.5 ± 8.0 kg/m^2^	1-week VLCD (800 kcal/day)	2-week VLCD (800 kcal/day)	NR

**Surgery abbreviations:** LSG (laparoscopic sleeve gastrectomy), LRYGB (laparoscopic Roux-en-Y gastric bypass), RYGB (Roux-en-Y gastric bypass), Lap-GBP (Laparoscopic Gastric Bypass). **Dietary terms:** VLCD (very low-calorie diet), LCD (low-calorie diet), VLCKD (very low-calorie ketogenic diet), VLCKD-SDM (very low-calorie ketogenic diet-specific diet model), O3FA (Omega-3 fatty acids), IMN (immunonutrition), PCD (portion-controlled diet), UDC (usual dietary care). **Comorbidities:** DM (diabetes mellitus), T2DM (type 2 diabetes mellitus), OSA (obstructive sleep apnea), NAFLD (non-alcoholic fatty liver disease), GERD (gastroesophageal reflux disease), PCOS (polycystic ovary syndrome), NR (not reported). **Other abbreviations**: BMI (body mass index), NR (not reported).

## Supplementary Materials

The following supporting information can be downloaded at: https://www.mdpi.com/article/10.3390/nu17091533/s1, Table S1: Search strategy performed in the different databases; Figure S1: Risk of bias graph (RoB): review authors’ judgements about each risk of bias item presented as percentages across all included studies; Figure S2: Risk of bias summary (RoB): review authors’ judgements about each risk of bias item for each included study; Figure S3: Risk of bias graph (ROBINS-I): review authors’ judgements about each risk of bias item presented as percentages across all included studies; Figure S4: Risk of bias summary (ROBINS-I): review authors’ judgements about each risk of bias item for each included study; Table S2: Excluded studies; Table S3: Summary of findings table using the GRADE approach.
