# Supplementary material for: Effectiveness and Safety of Preoperative Nutritional Interventions on Surgical Outcomes in Patients Undergoing Metabolic and Bariatric Surgery: A Systematic Review and Meta-Analysis
Source: Nutrients. 2025 Apr 30;17(9):1533. doi: 10.3390/nu17091533 (PMC12073371; doi:10.3390/nu17091533)
Supplement: Supplementary file 1 [file nutrients-17-01533-s001.zip › nutrients-3571454-supplementary.pdf]

**Table S1: Search strategy performed in the different databases**

| Database | Search strategy |
|---|---|
| Medline/PubMed **(n= 1191)** | ((((((((("bariatric surger*"[Title/Abstract]) OR ("metabolic surger*"[Title/Abstract])) OR ("stomach stapling"[Title/Abstract])) OR ("gastroenterostomy"[Title/Abstract])) OR ("gastroplasty"[Title/Abstract])) OR ("gastric bypass"[Title/Abstract])) OR (("bariatric surgery"[MeSH Terms]))) AND ((((("preoperative"[Title/Abstract]) OR ("pre-operative"[Title/Abstract])) OR ("presurg*"[Title/Abstract])) OR ("pre-surg*"[Title/Abstract])) OR ("Preoperative Period"[MeSH Terms] OR "Preoperative Care"[MeSH Terms] OR "Preoperative Exercise"[MeSH Terms]))) AND ((((((((((("pharmacological intervention*"[Title/Abstract]) OR ("pharmacologic action*"[Title/Abstract])) OR ("drug therapy"[Title/Abstract])) OR ("pharmacotherapy"[Title/Abstract])) OR ("glucagon-like peptide-1 receptor agonists"[Title/Abstract])) OR ("GLP-1 receptor agonists"[Title/Abstract])) OR ("orlistat"[Title/Abstract])) OR ("anti-obesity agent*"[Title/Abstract])) OR ("antiobesity agent*"[Title/Abstract])) OR ("weight loss drugs"[Title/Abstract])) OR ((((("pharmacologic actions"[MeSH Terms]) OR ("drug therapy"[MeSH Terms])) OR ("orlistat"[MeSH Terms])) OR ("glucagon-like peptide-1 receptor agonists"[MeSH Terms])) OR ("anti-obesity agents"[MeSH Terms]))) OR ((((((((((("non-pharmacological intervention*"[Title/Abstract]) OR ("non-pharmacological action*"[Title/Abstract])) OR ("diet"[Title/Abstract])) OR ("exercise"[Title/Abstract])) OR ("behavioral therap*"[Title/Abstract])) OR ("behavioral intervention*"[Title/Abstract])) OR ("lifestyle modification*"[Title/Abstract])) OR ("combined approach*"[Title/Abstract])) OR ("gastric balloon*"[Title/Abstract])) OR ("intragastric balloon*"[Title/Abstract])) OR (((("diet, reducing"[MeSH Terms]) OR ("preoperative exercise"[MeSH Terms])) OR ("behavior therapy"[MeSH Terms])) OR ("gastric balloon"[MeSH Terms])))) |
| Epistemonikos **(n= 487)** | (title:((title:(bariatric surger*) OR abstract:(bariatric surger*)) OR (title:(metabolic surger*) OR abstract:(metabolic surger*)) OR (title:(stomach stapling) OR abstract:(stomach stapling)) OR (title:(gastroenterostomy) OR abstract:(gastroenterostomy)) OR (title:(gastroplasty) OR abstract:(gastroplasty)) OR (title:(gastric bypass) OR abstract:(gastric bypass))) OR abstract:((title:(bariatric surger*) OR abstract:(bariatric surger*)) OR (title:(metabolic surger*) OR abstract:(metabolic surger*)) OR (title:(stomach stapling) OR abstract:(stomach stapling)) OR |

| | |
|---|---|
| | (title:(gastroenterostomy) OR abstract:(gastroenterostomy)) OR (title:(gastroplasty) OR abstract:(gastroplasty)) OR (title:(gastric bypass) OR abstract:(gastric bypass)))) AND (title:((title:(preoperative) OR abstract:(preoperative)) OR (title:(pre-operative) OR abstract:(pre-operative)) OR (title:(presurg*) OR abstract:(presurg*)) OR (title:(pre-surg*) OR abstract:(pre-surg*))) OR abstract:((title:(preoperative) OR abstract:(preoperative)) OR (title:(pre-operative) OR abstract:(pre-operative)) OR (title:(presurg*) OR abstract:(presurg*)) OR (title:(pre-surg*) OR abstract:(pre-surg*)))) AND (title:((title:((title:(pharmacological intervention*) OR abstract:(pharmacological intervention*)) OR (title:(pharmacologic action*) OR abstract:(pharmacologic action*)) OR (title:(drug therapy) OR abstract:(drug therapy)) OR (title:(pharmacotherapy) OR abstract:(pharmacotherapy)) OR (title:(glucagon-like peptide-1 receptor agonists) OR abstract:(glucagon-like peptide-1 receptor agonists)) OR (title:(GLP-1 receptor agonists) OR abstract:(GLP-1 receptor agonists)) OR (title:(orlistat) OR abstract:(orlistat)) OR (title:(anti-obesity agent*) OR abstract:(anti-obesity agent*)) OR (title:(antiobesity agent*) OR abstract:(antiobesity agent*)) OR (title:(weight loss drugs) OR abstract:(weight loss drugs))) OR abstract:((title:(pharmacological intervention*) OR abstract:(pharmacological intervention*)) OR (title:(pharmacologic action*) OR abstract:(pharmacologic action*)) OR (title:(drug therapy) OR abstract:(drug therapy)) OR (title:(pharmacotherapy) OR abstract:(pharmacotherapy)) OR (title:(glucagon-like peptide-1 receptor agonists) OR abstract:(glucagon-like peptide-1 receptor agonists)) OR (title:(GLP-1 receptor agonists) OR abstract:(GLP-1 receptor agonists)) OR (title:(orlistat) OR abstract:(orlistat)) OR (title:(anti-obesity agent*) OR abstract:(anti-obesity agent*)) OR (title:(antiobesity agent*) OR abstract:(antiobesity agent*)) OR (title:(weight loss drugs) OR abstract:(weight loss drugs)))) OR (title:((title:(non-pharmacological intervention*) OR abstract:(non-pharmacological intervention*)) OR (title:(non-pharmacological action*) OR abstract:(non-pharmacological action*)) OR (title:(diet) OR abstract:(diet)) OR (title:(exercise) OR abstract:(exercise)) OR (title:(behavioral therap*) OR abstract:(behavioral therap*)) OR (title:(behavioral intervention*) OR abstract:(behavioral intervention*)) OR (title:(lifestyle modification*) OR abstract:(lifestyle modification*)) OR (title:(combined approach*) OR abstract:(combined approach*)) OR (title:(gastric balloon*) OR abstract:(gastric balloon*)) OR (title:(intragastric balloon*) OR abstract:(intragastric balloon*))) OR abstract:((title:(non-pharmacological intervention*) OR abstract:(non- |

| | |
|---|---|
| | pharmacological intervention*)) OR (title:(non-pharmacological action*) OR abstract:(non-pharmacological action*)) OR (title:(diet) OR abstract:(diet)) OR (title:(exercise) OR abstract:(exercise)) OR (title:(behavioral therap*) OR abstract:(behavioral therap*)) OR (title:(behavioral intervention*) OR abstract:(behavioral intervention*)) OR (title:(lifestyle modification*) OR abstract:(lifestyle modification*)) OR (title:(combined approach*) OR abstract:(combined approach*)) OR (title:(gastric balloon*) OR abstract:(gastric balloon*)) OR (title:(intragastric balloon*) OR abstract:(intragastric balloon*))))) OR abstract:((title:((title:(pharmacological intervention*) OR abstract:(pharmacological intervention*)) OR (title:(pharmacologic action*) OR abstract:(pharmacologic action*)) OR (title:(drug therapy) OR abstract:(drug therapy)) OR (title:(pharmacotherapy) OR abstract:(pharmacotherapy)) OR (title:(glucagon-like peptide-1 receptor agonists) OR abstract:(glucagon-like peptide-1 receptor agonists)) OR (title:(GLP-1 receptor agonists) OR abstract:(GLP-1 receptor agonists)) OR (title:(orlistat) OR abstract:(orlistat)) OR (title:(anti-obesity agent*) OR abstract:(anti-obesity agent*)) OR (title:(antiobesity agent*) OR abstract:(antiobesity agent*)) OR (title:(weight loss drugs) OR abstract:(weight loss drugs))) OR abstract:((title:(pharmacological intervention*) OR abstract:(pharmacological intervention*)) OR (title:(pharmacologic action*) OR abstract:(pharmacologic action*)) OR (title:(drug therapy) OR abstract:(drug therapy)) OR (title:(pharmacotherapy) OR abstract:(pharmacotherapy)) OR (title:(glucagon-like peptide-1 receptor agonists) OR abstract:(glucagon-like peptide-1 receptor agonists)) OR (title:(GLP-1 receptor agonists) OR abstract:(GLP-1 receptor agonists)) OR (title:(orlistat) OR abstract:(orlistat)) OR (title:(anti-obesity agent*) OR abstract:(anti-obesity agent*)) OR (title:(antiobesity agent*) OR abstract:(antiobesity agent*)) OR (title:(weight loss drugs) OR abstract:(weight loss drugs)))) OR (title:((title:(non-pharmacological intervention*) OR abstract:(non-pharmacological intervention*)) OR (title:(non-pharmacological action*) OR abstract:(non-pharmacological action*)) OR (title:(diet) OR abstract:(diet)) OR (title:(exercise) OR abstract:(exercise)) OR (title:(behavioral therap*) OR abstract:(behavioral therap*)) OR (title:(behavioral intervention*) OR abstract:(behavioral intervention*)) OR (title:(lifestyle modification*) OR abstract:(lifestyle modification*)) OR (title:(combined approach*) OR abstract:(combined approach*)) OR (title:(gastric balloon*) OR abstract:(gastric balloon*)) OR (title:(intragastric balloon*) OR abstract:(intragastric balloon*))) OR abstract:((title:(non- |

| | |
|---|---|
| | pharmacological intervention*) OR abstract:(non-pharmacological intervention*)) OR (title:(non-pharmacological action*) OR abstract:(non-pharmacological action*)) OR (title:(diet) OR abstract:(diet)) OR (title:(exercise) OR abstract:(exercise)) OR (title:(behavioral therap*) OR abstract:(behavioral therap*)) OR (title:(behavioral intervention*) OR abstract:(behavioral intervention*)) OR (title:(lifestyle modification*) OR abstract:(lifestyle modification*)) OR (title:(combined approach*) OR abstract:(combined approach*)) OR (title:(gastric balloon*) OR abstract:(gastric balloon*)) OR (title:(intragastric balloon*) OR abstract:(intragastric balloon*)))))) |
| Cochrane CENTRAL<br>**(n = 775)** | ID Search Hits<br>#1 (bariatric surger*):ti,ab,kw OR (metabolic surger*):ti,ab,kw OR (gastric bypass):ti,ab,kw OR (gastroenterostomy):ti,ab,kw OR (gastroplasty):ti,ab,kw 8775<br>#2 MeSH descriptor: [Bariatric Surgery] explode all trees 1874<br>#3 #1 OR #2 8927<br>#4 (pre-operative):ti,ab,kw OR (presurg*):ti,ab,kw OR (pre-surg*):ti,ab,kw OR (preoperative):ti,ab,kw 52388<br>#5 MeSH descriptor: [Preoperative Period] explode all trees 521<br>#6 MeSH descriptor: [Preoperative Care] explode all trees 7304<br>#7 MeSH descriptor: [Preoperative Exercise] explode all trees 140<br>#8 #4 OR #5 OR #6 OR #753926<br>#9 #3 AND #8 1456<br>#10 (pharmacological intervention*):ti,ab,kw OR (pharmacologic action*):ti,ab,kw OR (drug therapy):ti,ab,kw OR (pharmacotherapy):ti,ab,kw OR (weight loss drugs):ti,ab,kw 579760<br>#11 (glucagon-like peptide-1 receptor agonists):ti,ab,kw OR (GLP-1 receptor agonists):ti,ab,kw OR (orlistat):ti,ab,kw OR (anti-obesity agent*):ti,ab,kw OR (antiobesity agent*):ti,ab,kw 2234<br>#12 MeSH descriptor: [Pharmacologic Actions] explode all trees 320599<br>#13 MeSH descriptor: [Drug Therapy] explode all trees 187621<br>#14 MeSH descriptor: [Orlistat] explode all trees 350<br>#15 MeSH descriptor: [Glucagon-Like Peptide-1 Receptor Agonists] explode all trees 36<br>#16 MeSH descriptor: [Anti-Obesity Agents] explode all trees 1059 |

| | |
|---|---|
| | #17 #10 OR #11 OR #12 OR #13 OR #14 OR #15 OR #16 708162<br>#18 (non-pharmacological intervention*):ti,ab,kw OR (non-pharmacological action*):ti,ab,kw OR (diet):ti,ab,kw OR (exercise):ti,ab,kw OR (behavioral therap*):ti,ab,kw 250183<br>#19 (behavioral intervention*):ti,ab,kw OR (lifestyle modification*):ti,ab,kw OR (combined approach*):ti,ab,kw OR (gastric balloon*):ti,ab,kw OR (intragastric balloon*):ti,ab,kw 66082<br>#20 MeSH descriptor: [Diet, Reducing] explode all trees 2503<br>#21 MeSH descriptor: [Preoperative Exercise] explode all trees 140<br>#22 MeSH descriptor: [Behavior Therapy] explode all trees 25737<br>#23 MeSH descriptor: [Gastric Balloon] explode all trees 75<br>#24 #18 OR 19 OR #20 OR #21 OR #22 OR #23 467991<br>#25 #17 OR #24 1022506<br>#26 #9 AND #25 775 |
| LILACS<br>**(n=751)** | (((bariatric surger*) OR (metabolic surger*) OR (stomach stapling) OR (gastroenterostomy) OR (gastroplasty) OR (gastric bypass) OR (mh:(bariatric surgery)))) AND (((preoperative) OR (pre-operative) OR (presurg*) OR (pre-surg*) OR (mh:(preoperative period)) OR (mh:(preoperative care)) OR (mh:(preoperative exercise)))) AND (((pharmacological intervention*) OR (pharmacologic action*) OR (drug therapy) OR (pharmacotherapy) OR (glucagon-like peptide-1 receptor agonists) OR (glp-1 receptor agonists) OR (orlistat) OR (anti-obesity agent*) OR (antiobesity agent*) OR (weight loss drugs) OR (mh:(pharmacologic actions)) OR (mh:(drug therapy)) OR (mh:(orlistat)) OR (mh:(glucagon-like peptide-1 receptor agonists)) OR (mh:(anti-obesity agents))) OR ((non-pharmacological intervention*) OR (non-pharmacological action*) OR (diet) OR (exercise) OR (behavioral therap*) OR (behavioral intervention*) OR (lifestyle modification*) OR (combined approach*) OR (gastric balloon*) OR (intragastric balloon*) OR (mh:(diet, reducing)) OR (mh:(preoperative exercise)) OR (mh:(behavior therapy)) OR (mh:(gastric balloon)))) AND instance:"lilacsplus" |
| BVS<br>**(n = 410)** | (((bariatric surger*) OR (metabolic surger*) OR (stomach stapling) OR (gastroenterostomy) OR (gastroplasty) OR (gastric bypass) OR (mh:(bariatric surgery)))) AND (((preoperative) OR (pre-operative) OR (presurg*) OR (pre-surg*) OR (mh:(preoperative period)) OR (mh:(preoperative care)) OR (mh:(preoperative exercise)))) AND (((pharmacological intervention*) OR (pharmacologic action*) OR (drug therapy) OR (pharmacotherapy) OR (glucagon-like peptide-1 receptor |

| | |
|---|---|
| | agonists) OR (glp-1 receptor agonists) OR (orlistat) OR (anti-obesity agent*) OR (antiobesity agent*) OR (weight loss drugs) OR (mh:(pharmacologic actions)) OR (mh:(drug therapy)) OR (mh:(orlistat)) OR (mh:(glucagon-like peptide-1 receptor agonists)) OR (mh:(anti-obesity agents))) OR ((non-pharmacological intervention*) OR (non-pharmacological action*) OR (diet) OR (exercise) OR (behavioral therap*) OR (behavioral intervention*) OR (lifestyle modification*) OR (combined approach*) OR (gastric balloon*) OR (intragastric balloon*) OR (mh:(diet, reducing)) OR (mh:(preoperative exercise)) OR (mh:(behavior therapy)) OR (mh:(gastric balloon)))) AND db:("LILACS" OR "IBECS" OR "WPRIM" OR "BINACIS" OR "BDENF" OR "CUMED" OR "BIGG" OR "SES-SP") AND instance:"regional" |
| Scopus **(n= 20)** | ((TITLE-ABS-KEY("bariatric surgery" OR "metabolic surgery") OR TITLE-ABS-KEY("gastric bypass" OR "gastroenterostomy" OR "gastroplasty"))) AND ((TITLE-ABS-KEY("preoperative weight loss" OR "pre-operative preparation" OR "preoperative optimization" OR "presurgical weight loss" OR "pre-surgical preparation"))) AND (((TITLE-ABS-KEY("preoperative pharmacotherapy" OR "preoperative medication") OR TITLE-ABS-KEY("GLP-1 receptor agonists" OR "glucagon-like peptide-1 receptor agonists") OR TITLE-ABS-KEY("preoperative orlistat" OR "preoperative anti-obesity medication"))) OR ((TITLE-ABS-KEY("preoperative diet" OR "pre-surgical diet") OR TITLE-ABS-KEY("preoperative lifestyle intervention" OR "presurgical lifestyle modification") OR TITLE-ABS-KEY("preoperative behavioral therapy" OR "pre-surgical exercise program")))) |
| Google scholar **(n= 516)** | allintitle: ("bariatric surgery" OR "metabolic surgery" OR "gastric bypass") AND ("preoperative" OR "pre-operative" OR "presurgical") |

**Figure S1: Risk of bias graph (RoB): review authors' judgements about each risk of bias item presented as percentages across all included studies**

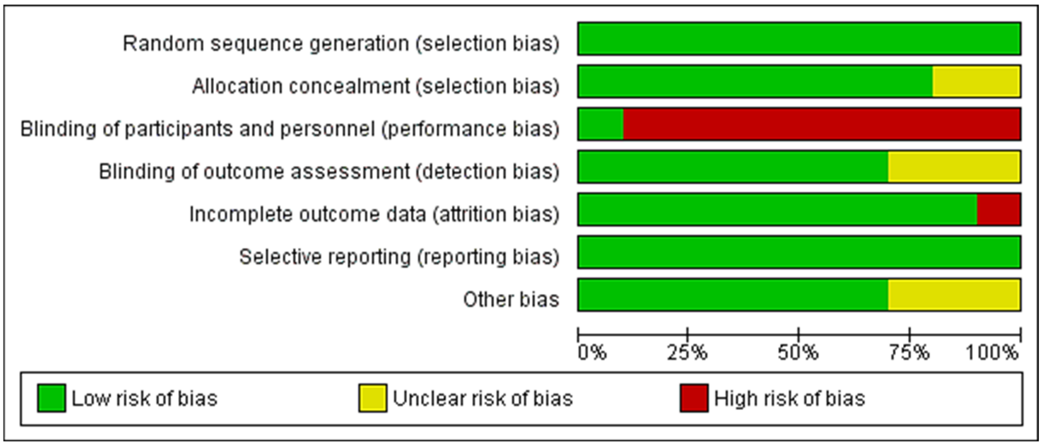


**Figure S2: Risk of bias summary (RoB): review authors' judgements about each risk of bias item for each included study**

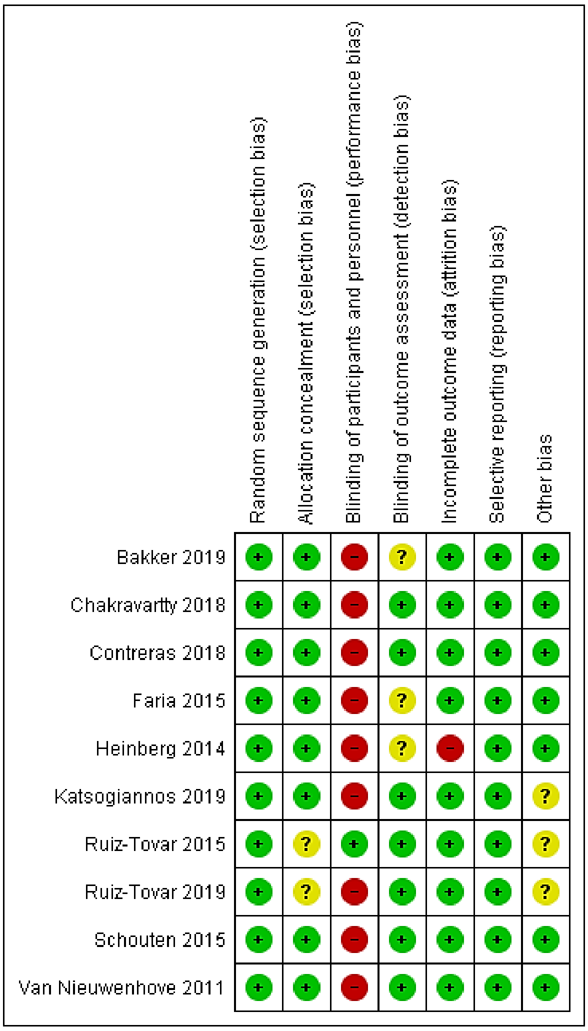

**Figure S3: Risk of bias graph (ROBINS-I): review authors' judgements about each risk of bias item presented as percentages across all included studies**

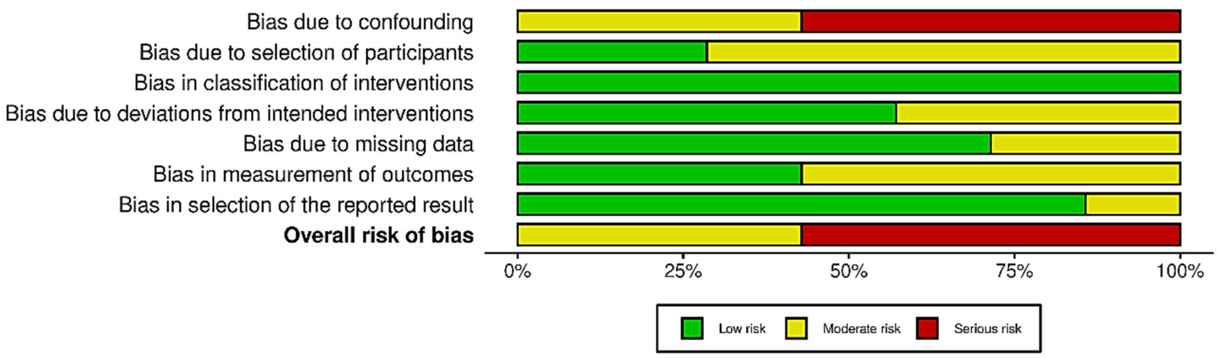


**Figure S4: Risk of bias summary (ROBINS-I): review authors' judgements about each risk of bias item for each included study**.

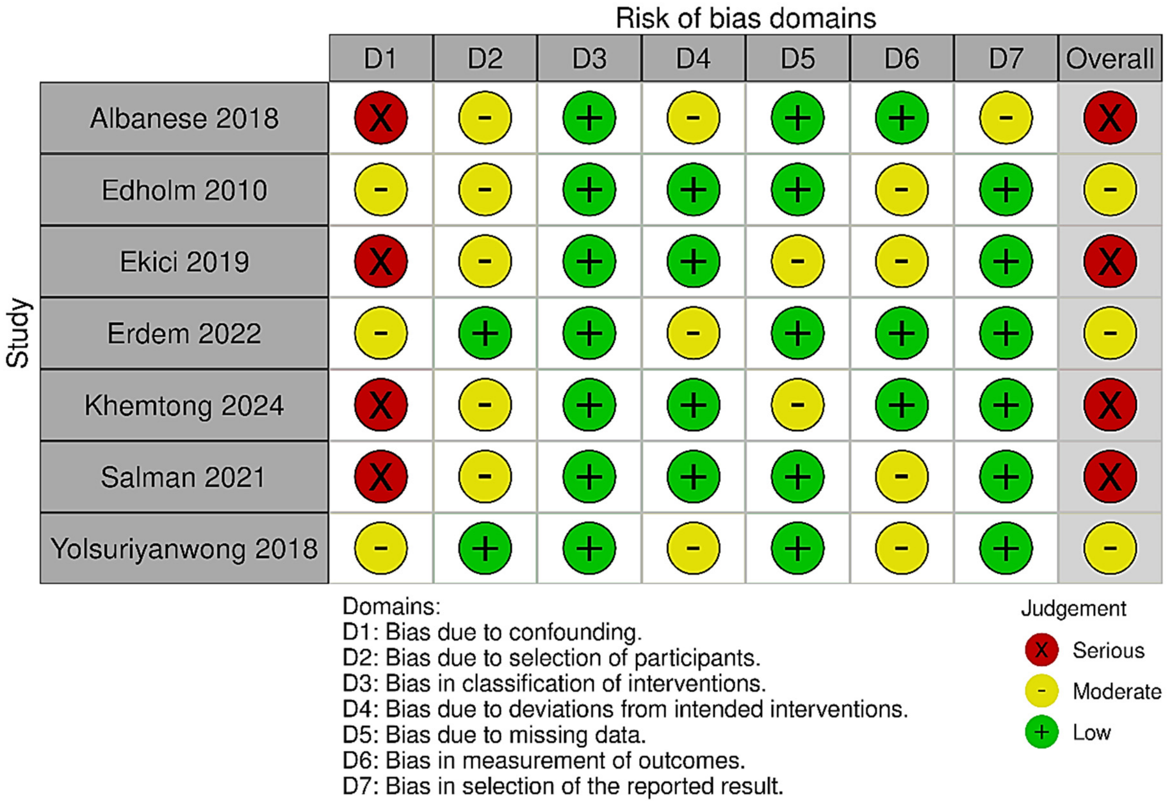

**Table S2: Excluded studies**

| # | Ref | Year | Reference | Exclusion Reason |
|---|---|---|---|---|
| 1 | [62] | 2024 | Mangarelli C, Fell G, Hobbs E, Lowry KW, Williams E, Pratt JSA. Pediatric metabolic and bariatric surgery: indications and preoperative multidisciplinary evaluation. Surgery for Obesity and Related Diseases. 2024;20: 1334–1342. | Wrong population |
| 2 | [63] | 2024 | Antonythasan R. Association Between Appetite-Regulating Hormonal Levels and Body Composition Among Adults With Pre-operative Bed Undergoing Bariatric Surgery: A Prospective Cohort Study Protocol. Undergraduate Research in Natural and Clinical Science and Technology Journal. 2024;8: 1–12. | Wrong intervention |
| 3 | [64] | 2024 | Lucocq J, Homyer K, Geropoulos G, Thakur V, Stansfield D, Joyce B, et al. Long-Term Weight Loss and Comorbidity Resolution of Laparoscopic Sleeve Gastrectomy and Laparoscopic Roux-en-Y Gastric Bypass and the Impact of Preoperative Weight Loss on Overall Outcome. Surg Laparosc Endosc Percutan Tech. 2024;34: 466–471. | Wrong intervention |
| 4 | [65] | 2024 | Shinder E, Hanson P, Phillips H, Tuppo C, Spaniolas K, Pryor A, et al. Preoperative medically supervised weight loss programs and weight loss outcomes following bariatric surgery–a prospective analysis. Surgery for Obesity and Related Diseases. 2024;20: 165–172. | Wrong outcome |
| 5 | [66] | 2024 | Goday A, Bagán A, Casajoana A, Serra C, Pera M, Villatoro M, et al. Effects of Preoperative Quadruple Therapy for Helicobacter pylori on Bariatric Surgery Metabolic Outcomes. Obes Surg. 2024;34: 1196–1206. doi:10.1007/s11695-024-07091-x | Wrong outcome |
| 6 | [67] | 2024 | Aeschbacher P, Garcia A, Dourado J, Rogers P, Zoe G, Pena A, et al. Outcome of gastric electrical stimulator with and without pyloromyotomy for refractory gastroparesis. Surg Endosc. 2024;38: 6026–6032. doi:10.1007/s00464-024-11099-w | Wrong outcome |
| 7 | [68] | 2024 | Hany M, Shafei M El, Ibrahim M, Agayby ASS, Abouelnasr AA, Aboelsoud MR, et al. The role of preoperative abdominal ultrasound in the preparation of patients undergoing primary metabolic and bariatric surgery: a machine learning algorithm on 4418 patients' records. Obes Surg. 2024;34: 3445–3458. | Wrong outcome |
| 8 | [69] | 2024 | Rodrigues J, Magalhães V, Santos MP, Reis C, Pichel F, Soares P, et al. Weight loss in patients with severe obesity after bariatric surgery-the potential role of the chrono-nutrition, chronotype and the circadian misalignment: A study protocol of the ChronoWise prospective cohort. PLoS One. 2024;19: e0313096. doi:10.1371/journal.pone.0313096 | Wrong outcome |
| 9 | [70] | 2024 | NCT06480708. Evaluation of the Ketogenic Diet to Improve Post Operative Cognitive Decline in Cardiac Surgery. 2024. Available: https://www.cochranelibrary.com/central/doi/10.1002/central/CN-02733146/full | Protocol |
| 10 | [71] | 2024 | NCT06461247. Compliance With Standard Care vs Ready to Eat Meals Prior to Bariatric Surgery. 2024. Available: https://www.cochranelibrary.com/central/doi/10.1002/central/CN-02709336/full | Protocol |
| 11 | [72] | 2024 | MacCormick A, Puckett M, Aroori S. The safety, tolerability and clinical impact of pre-operative very low-calorie diet prior to non-bariatric abdominal surgery: a systematic review. Langenbecks Arch Surg. 2024;409: 327. doi:10.1007/s00423-024-03509-3 | Without comparison |
| 12 | [73] | 2024 | Griffin SB, Palmer MA, Strodl E, Lai R, Guo C, Chuah TL, et al. Impact of a dietitian-led very low calorie diet clinic on perioperative risk for patients with obesity awaiting elective, non-bariatric surgery: A retrospective cohort study. Surgery. 2024;175: 463–470. doi:10.1016/j.surg.2023.09.047 | Without comparison |
| 13 | [74] | 2024 | Hassan M, Barajas-Gamboa JS, Kanwar O, T L-SJ, Tannous D, Corcelles R, et al. The role of dietitian follow-ups on nutritional outcomes post-bariatric surgery. Surg Obes Relat Dis. 2024;20: 407–412. doi:10.1016/j.soard.2023.10.017 | Without comparison |
| 14 | [75] | 2024 | Marek RJ, Heinberg LJ. Should presurgical psychological evaluations still be a mandated requirement for metabolic and bariatric surgery? A critique of the literature and thoughts on future directions. Surgery for Obesity and Related Diseases. 2024. | Wrong type publication |
| 15 | [76] | 2024 | Lau LCM, Chan PK, Lui TWD, Choi SW, Au E, Leung T, et al. Preoperative weight loss interventions before total hip and knee arthroplasty: a systematic review of randomized controlled trials. Arthroplasty (London, England). 2024;6: 30. doi:10.1186/s42836-024-00252-4 | Wrong type publication |

| | | | | |
|---|---|---|---|---|
| 16 | [77] | 2024 | Chowdhury N, Hasnan S, Ullah S, Thompson SK. Low-calorie diets are effective for weight loss in patients undergoing benign upper gastrointestinal surgery: a systematic review and meta-analysis. Surg Endosc. 2024;38: 4171–4185. doi:10.1007/s00464-024-11016-1 | Wrong type publication |
| 17 | [78] | 2024 | MacCormick A, Puckett M, Aroori S. The safety, tolerability and clinical impact of pre-operative very low-calorie diet prior to non-bariatric abdominal surgery: a systematic review. Langenbecks Arch Surg. 2024;409: 327. doi:10.1007/s00423-024-03509-3 | Wrong type publication |
| 18 | [79] | 2024 | Maheta B, Shehabat M, Khalil R, Wen J, Karabala M, Manhas P, et al. The Effectiveness of Patient Education on Laparoscopic Surgery Postoperative Outcomes to Determine Whether Direct Coaching Is the Best Approach: Systematic Review of Randomized Controlled Trials. JMIR Perioper Med. 2024;7: e51573. doi:10.2196/51573 | Wrong type publication |
| 19 | [80] | 2024 | Khalooeifard R, Rahmani J, Ghoreishy SM, Tavakoli A, Najjari K, Talebpour M. Evaluate the Effects of Different Types of Preoperative Restricted Calorie Diets on Weight, Body Mass Index, Operation Time and Hospital Stay in Patients Undergoing Bariatric Surgery: a Systematic Review and Meta Analysis Study. Obes Surg. 2024;34: 236–249. doi:10.1007/s11695-023-06973-w | Wrong type publication |
| 20 | [81] | 2024 | Rodrigues J, Magalhães V, Santos MP, Reis C, Pichel F, Soares P, et al. Weight loss in patients with severe obesity after bariatric surgery-the potential role of the chrono-nutrition, chronotype and the circadian misalignment: A study protocol of the ChronoWise prospective cohort. PLoS One. 2024;19: e0313096. doi:10.1371/journal.pone.0313096 | Wrong type publication |
| 21 | [82] | 2024 | Karpińska I, Dowgiałło-Gornowicz N, Franczak P, Orłowski M, Lech P, Kowalski G, et al. Factors contributing to weight loss success after bariatric procedures in patient over 65 years old: a multicenter retrospective cohort study. Int J Surg. 2024;110: 4893–4899. doi:10.1097/JS9.0000000000001588 | Wrong type publication |
| 22 | [83] | 2024 | Hage K, Abi Mosleh K, Sample JW, Vierkant RA, Mundi MS, Spaniolas K, et al. Preoperative duration of type 2 diabetes mellitus and remission after Roux-en-Y gastric bypass: A single center long-term cohort study. International Journal of Surgery. 2024; 10.1097. | Wrong type publication |
| 23 | [84] | 2024 | Griffin SB, Palmer MA, Strodl E, Lai R, Guo C, Chuah TL, et al. Impact of a dietitian-led very low calorie diet clinic on perioperative risk for patients with obesity awaiting elective, non-bariatric surgery: A retrospective cohort study. Surgery. 2024;175: 463–470. doi:10.1016/j.surg.2023.09.047 | Wrong type publication |
| 24 | [85] | 2024 | Yang N, Hua H, Liu S, Zhang S, Zhao X, Zhang P, et al. The current status and challenges of perioperative management of patients with a BMI of greater than or equal to 50 kg/m 2 undergoing bariatric surgery in China: a multicenter cross-sectional study. Int J Surg. 2024;110: 2577–2582. doi:10.1097/JS9.0000000000001108 | Wrong type publication |
| 25 | [86] | 2024 | Nogueira PLB, Coimbra de Paula C, Dock-Nascimento DB, Aguilar-Nascimento JE. Metabolic effects of an oral carbohydrate-whey protein supplement after fasting in volunteers: a randomized controlled crossover trial. 2024;117: 112251. doi:10.1016/j.nut.2023.112251 | Wrong type publication |
| 26 | [87] | 2024 | Zhu Y, Zhang Q, Chen Y, Gong Y. Letter to the Editor about “Preoperative duration of type 2 diabetes mellitus and remission after Roux-en-Y gastric bypass: A single center long-term cohort study.” International Journal of Surgery. 2024; 10.1097. | Wrong type publication |
| 27 | [88] | 2024 | Diep C, Lee S, Xue Y, Xiao M, Pivetta B, Daza JF, et al. Preoperative depression and outcomes after metabolic and bariatric surgery: A systematic narrative review. Obesity Reviews. 2024; e13743. | Wrong type publication |
| 28 | [89] | 2024 | Lucocq J, Homyer K, Geropoulos G, Thakur V, Stansfield D, Joyce B, et al. Long-Term Weight Loss and Comorbidity Resolution of Laparoscopic Sleeve Gastrectomy and Laparoscopic Roux-en-Y Gastric Bypass and the Impact of Preoperative Weight Loss on Overall Outcome. Surg Laparosc Endosc Percutan Tech. 2024;34: 466–471. | Wrong type publication |
| 29 | [90] | 2023 | Muir D, Choi B, Holden M, Clements C, Stevens J, Ratnasingham K, et al. Preoperative oesophagogastroduodenoscopy and the effect on bariatric surgery: a systematic review and meta-analysis. Obes Surg. 2023;33: 2546–2556. | Wrong population |
| 30 | [91] | 2023 | Hassan M, Barajas-Gamboa JS, Kanwar O, T L-SJ, Tannous D, Corcelles R, et al. The role of dietitian follow-ups on nutritional outcomes post-bariatric surgery. Surg Obes Relat Dis. 2024;20: 407–412. doi:10.1016/j.soard.2023.10.017 | Wrong population |

| | | | | |
|---|---|---|---|---|
| 31 | [92] | 2023 | Strauss AL, Triggs JR, Tewksbury CM, Soriano I, Wernsing DS, Dumon KR, et al. Conversion to Roux-En-Y Gastric Bypass: a successful means of mitigating reflux after laparoscopic sleeve gastrectomy. Surg Endosc. 2023;37: 5374–5379. doi:10.1007/s00464-023-10024-x | Wrong population |
| 32 | [93] | 2023 | Ho C, Samwil SNM, Kahairudin Z, Jamhuri N, A AA. Exercise and pre-habilitation with high whey-protein-based meal replacement therapy promote weight loss and preserve muscle mass before bariatric surgery. Asian J Surg. 2023;46: 3716–3721. doi:10.1016/j.asjsur.2023.03.026 | Wrong intervention |
| 33 | [94] | 2023 | Martines G, Dezi A, Giove C, Lantone V, Rotelli MT, Picciariello A, et al. Efficacy of Intragastric Balloon versus Liraglutide as Bridge to Surgery in Super-Obese Patients. Obes Facts. 2023;16: 457–464. doi:10.1159/000531459 | Wrong intervention |
| 34 | [95] | 2023 | Lo HC, Hsu SC. Effectiveness of a preoperative orlistat-based weight management plan and its impact on the results of one-anastomosis gastric bypass: A retrospective study. PLoS One. 2023;18: e0289006. doi:10.1371/journal.pone.0289006 | Wrong intervention |
| 35 | [96] | 2023 | Al-Shehri SS, Alilyyani B, Alshareef K, Shami AA, Alshehri A, Alghamdi A, et al. Evaluation of preoperative dyslipidemia and micronutrient status in obese patients undergoing bariatric surgery in Taif, Saudi Arabia: A retrospective study. Obes Med. 2023;39: 100486. | Wrong intervention |
| 36 | [97] | 2023 | Torensma B, Hany M, Bakker MJS, M van V, A I 't VB, Dahan A, et al. Cross-sectional E-survey on the Incidence of Pre- and Postoperative Chronic Pain in Bariatric Surgery. Obes Surg. 2023;33: 204–210. doi:10.1007/s11695-022-06354-9 | Wrong outcome |
| 37 | [98] | 2023 | Sadhai P, Coetzee A, Conradie-Smit M, Greyling CJ, R van G, I du T, et al. Nutritional deficiency in South African adults scheduled for bariatric surgery. Front Endocrinol (Lausanne). 2023;14: 1120531. doi:10.3389/fendo.2023.1120531 | Wrong outcome |
| 38 | [99] | 2023 | NCT05918471. Preoperative very low energy diets for obese patients Undergoing Non-bariatric Surgery. 2023. Available: https://www.cochranelibrary.com/central/doi/10.1002/central/CN-02577708/full | Protocol |
| 39 | [100] | 2023 | NCT05717595. BaRiatric Surgery AnD FRUctose Handeling In Obese subjecTs. 2023. Available: https://www.cochranelibrary.com/central/doi/10.1002/central/CN-02522777/full | Protocol |
| 40 | [101] | 2023 | Barrea L, Verde L, Schiavo L, Sarno G, Camajani E, Iannelli A, et al. Very Low-Calorie Ketogenic Diet (VLCKD) as Pre-Operative First-Line Dietary Therapy in Patients with Obesity Who Are Candidates for Bariatric Surgery. Nutrients. 2023;15. doi:10.3390/nu15081907 | Without comparison |
| 41 | [102] | 2023 | Abbott S, Price C, Pournaras DJ, Coulman K. Variation and outcomes of liver-reducing dietary regimens before bariatric surgery: a national retrospective cohort study. Surg Obes Relat Dis. 2023;19: 102–108. doi:10.1016/j.soard.2022.10.027 | Without comparison |
| 42 | [103] | 2023 | Diab S, Bertin JB, Simeu B, Rohr S, Brigand C, Deharvengt C, et al. Impact of Preoperative Protein Sparing Modified Fast Diet on Bariatric Surgery. Obes Surg. 2023;33: 17–24. doi:10.1007/s11695-022-06361-w | Without comparison |
| 43 | [104] | 2023 | Hosseini-Esfahani F, Kazemi-Aliakbar M, Koochakpoor G, Barzin M, Khalaj A, Valizadeh M, et al. Diet quality and anthropometric indices of patients undergone bariatric surgery: the prospective Tehran obesity treatment study. BMC Surg. 2023;23: 125. doi:10.1186/s12893-023-02032-4 | Without comparison |
| 44 | [105] | 2023 | Castaldo G, Schiavo L, Pagano I, Molettieri P, Conte A, Sarno G, et al. Clinical Impact of Enteral Protein Nutritional Therapy on Patients with Obesity Scheduled for Bariatric Surgery: A Focus on Safety, Efficacy, and Pathophysiological Changes. Nutrients. 2023;15. doi:10.3390/nu15061492 | Wrong type publication |
| 45 | [106] | 2023 | Musallam R, Rahman SH, Shweikeh F, Beran A, Mohan B, Daaboul R, et al. S520 Esophagogastroduodenoscopy Plays an Important Preoperative Role in Bariatric Surgery: Systematic Review and Meta-Analysis. Official journal of the American College of Gastroenterology ǀ ACG. 2023;118: S378. | Wrong type publication |
| 46 | [107] | 2023 | Nasser K, Verhoeff K, Mocanu V, Kung JY, Purich K, Switzer NJ, et al. New persistent opioid use after bariatric surgery: a systematic review and pooled proportion meta-analysis. Surg Endosc. 2023;37: 703–714. doi:10.1007/s00464-022-09291-x | Wrong type publication |

| | | | | |
|---|---|---|---|---|
| 47 | [108] | 2023 | Patel PH, Ho T, Upadhyay SM. A Systematic Review of Warfarin Use in Post-Bariatric Surgery Patients: Cases Compiled From a Literature Review. Ann Pharmacother. 2023;57: 193–197. doi:10.1177/10600280221105312 | Wrong type publication |
| 48 | [109] | 2023 | Almuhtadi Y, Alageel S. Systematic Review of Mindfulness-Based Interventions for Weight Management Among Pre- and Post-bariatric Surgery Patients. Adv Mind Body Med. 2023;37: 15–22. Available: https://pubmed.ncbi.nlm.nih.gov/38345771/ | Wrong type publication |
| 49 | [110] | 2023 | Giustina A, L di F, Facciorusso A, Adler RA, Binkley N, Bollerslev J, et al. Vitamin D status and supplementation before and after Bariatric Surgery: Recommendations based on a systematic review and meta-analysis. Rev Endocr Metab Disord. 2023;24: 1011–1029. doi:10.1007/s11154-023-09831-3 | Wrong type publication |
| 50 | [111] | 2023 | Gao Z, Liang Y, Huang S, Wu Z, Li M, Yang J. Prevalence and associated factors of vitamin D deficiency after Roux-en-Y gastric bypass: a systematic review and meta-analysis. Int J Surg. 2023;109: 4273–4285. doi:10.1097/JS9.0000000000000732 | Wrong type publication |
| 51 | [112] | 2023 | Hacker KS, Salwen-Deremer JK. Comment on: Re-evaluating the binge eating scale cut-off using DSM-5 criteria: analysis and replication in presurgical metabolic and bariatric surgery samples. Surgery for Obesity and Related Diseases. 2023;19: 950–951. | Wrong type publication |
| 52 | [113] | 2023 | Abbott S, Price C, Pournaras DJ, Coulman K. Variation and outcomes of liver-reducing dietary regimens before bariatric surgery: a national retrospective cohort study. Surg Obes Relat Dis. 2023;19: 102–108. doi:10.1016/j.soard.2022.10.027 | Wrong type publication |
| 53 | [114] | 2023 | Lenér F, Höskuldsdóttir G, Landin-Wilhelmsen K, Björkelund C, Eliasson B, Fändriks L, et al. Anaemia in patients with self-reported use of iron supplements in the BAriatric surgery SUbstitution and nutrition study: A prospective cohort study. Nutr Metab Cardiovasc Dis. 2023;33: 998–1006. doi:10.1016/j.numecd.2023.02.008 | Wrong type publication |
| 54 | [115] | 2023 | Torensma B, Hany M, Bakker MJS, M van V, A I 't VB, Dahan A, et al. Cross-sectional E-survey on the Incidence of Pre- and Postoperative Chronic Pain in Bariatric Surgery. Obes Surg. 2023;33: 204–210. doi:10.1007/s11695-022-06354-9 | Wrong type publication |
| 55 | [116] | 2023 | Maldonado FHR, Mega PF, Germano CW, Dias LLC, Callejas GH, Gestic MA, et al. Impact of pre-operative weight loss on non-alcoholic fatty liver disease histopathology and insulin resistance in individuals undergoing bariatric surgery: a propensity matched cross-sectional comparison. Sao Paulo Medical Journal. 2023;142: e2022663. | Wrong type publication |
| 56 | [117] | 2023 | Aderinto N, Olatunji G, Kokori E, Olaniyi P, Isarinade T, Yusuf IA. Recent advances in bariatric surgery: a narrative review of weight loss procedures. Ann Med Surg (Lond). 2023;85: 6091–6104. doi:10.1097/MS9.0000000000001472 | Wrong type publication |
| 57 | [118] | 2023 | Rojas R, Romani D, Guerrero H, Cruz D, Poggi L, Poggi L. Topic: Bariatrics As It Relates to Foregut Disease Abstract ID: 102 Laparoscopic Sleeve Gastrectomy Versus Roux-en-Y Gastric Bypass: Preoperative and Follow Up Findings in Dismotility and GERD Symptoms. Foregut. 2023;3: 429–430. | Wrong type publication |
| 58 | [119] | 2023 | Phan A, Hage M, Zaharia R, Vigan M, Coursault S, Wilson S, et al. Nutritional Status of Vegetarian Patients Before and After Bariatric Surgery: a Monocentric Retrospective Observational Case-Control Study. Obes Surg. 2023;33: 1356–1365. doi:10.1007/s11695-023-06538-x | Wrong type publication |
| 59 | [120] | 2023 | Elkhoury D, Elkhoury C, Gorantla VR. Improving Access to Child and Adolescent Weight Loss Surgery: A Review of Updated National and International Practice Guidelines. Cureus. 2023;15: e38117. doi:10.7759/cureus.38117 | Wrong type publication |
| 60 | [121] | 2022 | Lo HC, Hsu S-C. Is there a causal relationship between the consequences of preoperative weight control using orlistat-based intervention and the result after one-anastomosis gastric bypass? A retrospective study. 2022. | Wrong intervention |
| 61 | [122] | 2022 | Henning T, Kochlik B, Kusch P, Strauss M, Jurić V, Pignitter M, et al. Pre-operative assessment of micronutrients, amino acids, phospholipids and oxidative stress in bariatric surgery candidates. Antioxidants. 2022;11: 774. | Wrong intervention |
| 62 | [123] | 2022 | Hanson MN, Dennis S, Altieri MS, Andalib A. Reflux and bariatric surgery: a review of pre-operative assessment and post-operative approach. Mini-invasive Surg. 2022;6: 9. | Wrong outcome |
| 63 | [124] | 2022 | Martin-Fernandez KW, Creel DB, Schuh LM. Psychosocial and behavioral correlates of weight loss 12 to 15 years after bariatric surgery. J Behav Med. 2022;45: 252–259. doi:10.1007/s10865-021-00263-5 | Wrong outcome |

| | | | | |
|---|---|---|---|---|
| 64 | [125] | 2022 | Murtha JA, Venkatesh M, Liu N, Jawara D, Hanlon BM, Hanrahan LP, et al. Association between neighborhood food environments and bariatric surgery outcomes. Surg Obes Relat Dis. 2022;18: 1357–1364. doi:10.1016/j.soard.2022.08.007 | Wrong outcome |
| 65 | [126] | 2022 | Verdaguer M, Beisani M, López ÓG, Vilallonga R, de Gordejuela AGR, Jurado MJG, et al. Preoperative weight loss and postoperative short and long-term results in Bariatric Surgery. 2022. | Wrong outcome |
| 66 | [127] | 2022 | Zarshenas N, Tapsell LC, Batterham M, Neale EP, Talbot ML. Investigating the prevalence of nutritional abnormalities in patients prior to and following bariatric surgery. Nutr Diet. 2022;79: 590–601. doi:10.1111/1747-0080.12747 | Wrong outcome |
| 67 | [128] | 2022 | Childs J, Mudge LA, Esterman A, Thompson SK. What Is the Optimal Time on a Low-Calorie Diet Prior to Laparoscopic Anti-reflux Surgery? A Prospective Case-Controlled Study. Journal of Gastrointestinal Surgery. 2022;26: 2249–2254. doi:10.1007/s11605-022-05438-2 | Without comparison |
| 68 | [129] | 2022 | Lange UG, Moulla Y, Schütz T, Blüher M, Peter V, Shang E, et al. Effectiveness and Tolerability of a Two-Week Hypocaloric Protein-Rich Diet Prior to Obesity Surgery with Two Different Diet Interventions: a Prospective Randomized Trial. Obes Surg. 2022;32: 2903–2913. doi:10.1007/s11695-022-06180-z | Without comparison |
| 69 | [130] | 2022 | Meijer JL, Roderka MN, Chinburg EL, Renier TJ, McClure AC, Rothstein RI, et al. Alterations in Fecal Short-Chain Fatty Acids after Bariatric Surgery: Relationship with Dietary Intake and Weight Loss. Nutrients. 2022;14. doi:10.3390/nu14204243 | Without comparison |
| 70 | [131] | 2022 | University Hospital France S. Impact of Preoperative Hypocaloric Hyperproteinic Lipid Restricting Diet on Bariatric Surgery. clinicaltrials.gov. 2022. Available: http://www.epistemonikos.org/documents/67fcefe88d436484feb146114da7bc2ec6ced1d2 | Without comparison |
| 71 | [132] | 2022 | Colangeli L, Gentileschi P, Sbraccia P, Guglielmi V. Ketogenic Diet for Preoperative Weight Reduction in Bariatric Surgery: A Narrative Review. Nutrients. 2022;14. doi:10.3390/nu14173610 | Without comparison |
| 72 | [133] | 2022 | Gastaldo I, Casas R, Moizé V. Clinical Impact of Mediterranean Diet Adherence before and after Bariatric Surgery: A Narrative Review. Nutrients. 2022;14. doi:10.3390/nu14020393 | Without comparison |
| 73 | [134] | 2022 | Sasaki A, Umemura A, Ishida K, Takahashi N, Nikai H, Nitta H, et al. The Concept of Indeterminable NASH Inducted by Preoperative Diet and Metabolic Surgery: Analyses of Histopathological and Clinical Features. Biomedicines. 2022;10. doi:10.3390/biomedicines10020453 | Without comparison |
| 74 | [135] | 2022 | Mills J, Liebert C, Pratt J, Earley M, Eisenberg D. Complete telehealth for multidisciplinary preoperative workup does not delay time to metabolic and bariatric surgery: a pilot study. Obes Surg. 2022;32: 3605–3610. | Wrong type publication |
| 75 | [136] | 2022 | Smith NA, Martin G, Marginson B. Preoperative assessment and prehabilitation in patients with obesity undergoing non-bariatric surgery: A systematic review. J Clin Anesth. 2022;78: 110676. doi:10.1016/j.jclinane.2022.110676 | Wrong type publication |
| 76 | [137] | 2022 | Naik A, MacInnis B, McNicholas M, Arnold PM. 125. Impact of preoperative bariatric surgery on elective spine fusion and decompression: a systematic review and meta-analysis. The Spine Journal. 2022;22: S66–S67. | Wrong type publication |
| 77 | [138] | 2022 | Hung KC, Wu SC, Chiang MH, Hsu CW, Chen JY, Huang PW, et al. Analgesic Efficacy of Gabapentin and Pregabalin in Patients Undergoing Laparoscopic Bariatric Surgeries: a Systematic Review and Meta-analysis. Obes Surg. 2022;32: 2734–2743. doi:10.1007/s11695-022-06109-6 | Wrong type publication |
| 78 | [139] | 2022 | Ikesaka R, Kaur B, Crowther M, Rajasekhar A. Efficacy and safety of pre-operative insertion of inferior vena cava filter in patients undergoing bariatric surgery: a systematic review. J Thromb Thrombolysis. 2022;54: 502–523. | Wrong type publication |
| 79 | [140] | 2022 | Durey BJ, Fritche D, Martin DS, Best LMJ. The Effect of Pre-operative Exercise Intervention on Patient Outcomes Following Bariatric Surgery: a Systematic Review and Meta-analysis. Obes Surg. 2022;32: 160–169. doi:10.1007/s11695-021-05743-w | Wrong type publication |
| 80 | [141] | 2022 | Hart A, Sun Y, Titcomb TJ, Liu B, Smith JK, Correia MLG, et al. Association between preoperative serum albumin levels with risk of death and postoperative complications after bariatric surgery: a retrospective cohort study. Surgery for obesity and related diseases. 2022;18: 928–934. | Wrong type publication |

| | | | | |
|---|---|---|---|---|
| 81 | [142] | 2022 | Hider AM, Ehlers AP. Comment on: Association between preoperative serum albumin levels with risk of death and postoperative complications after bariatric surgery: a retrospective cohort study. Surgery for Obesity and Related Diseases. 2022;18: e51–e52. | Wrong type publication |
| 82 | [143] | 2022 | Fadel MG, Fehervari M, Lairy A, Das B, Alyaqout K, Ashrafian H, et al. Clinical outcomes of single-stage versus two-stage laparoscopic Roux-en-y gastric bypass in the management of obesity (BMI ≥ 50 kg/m(2)): a retrospective cohort study. Langenbecks Arch Surg. 2022;407: 3349–3356. doi:10.1007/s00423-022-02664-9 | Wrong type publication |
| 83 | [144] | 2022 | Nymo S, Lundanes J, Aukan M, Sandvik J, Johnsen G, Græslie H, et al. Diet and physical activity are associated with suboptimal weight loss and weight regain 10-15 years after Roux-en-Y gastric bypass: A cross-sectional study. Obes Res Clin Pract. 2022;16: 163–169. doi:10.1016/j.orcp.2022.03.006 | Wrong type publication |
| 84 | [145] | 2022 | Colangeli L, Gentileschi P, Sbraccia P, Guglielmi V. Ketogenic Diet for Preoperative Weight Reduction in Bariatric Surgery: A Narrative Review. Nutrients. 2022;14. doi:10.3390/nu14173610 | Wrong type publication |
| 85 | [146] | 2022 | Gastaldo I, Casas R, Moizé V. Clinical Impact of Mediterranean Diet Adherence before and after Bariatric Surgery: A Narrative Review. Nutrients. 2022;14. doi:10.3390/nu14020393 | Wrong type publication |
| 86 | [147] | 2022 | Baillot A, St-Pierre M, Lapointe J, Bernard P, Bond D, Romain AJ, et al. Acceptability and Feasibility of the Telehealth Bariatric Behavioral Intervention to Increase Physical Activity: Protocol for a Single-Case Experimental Study. JMIR Res Protoc. 2022;11: e39633. doi:10.2196/39633 | Wrong type publication |
| 87 | [148] | 2022 | Marchitelli S, Ricci E, Mazza C, Roma P, Tambelli R, Casella G, et al. Obesity and Psychological Factors Associated with Weight Loss after Bariatric Surgery: A Longitudinal Study. Nutrients. 2022;14. doi:10.3390/nu14132690 | Wrong type publication |
| 88 | [149] | 2021 | Abdelsalam AM, AMSE E, Salman MA, Nassef SA, Elfergany HM, Aisha HAA. Adding a Preoperative Dose of LMWH may Decrease VTE Following Bariatric Surgery. World J Surg. 2021;45: 126–131. doi:10.1007/s00268-020-05782-x | Wrong intervention |
| 89 | [150] | 2021 | Garcia-Delgado Y, Lopez-Madrazo-hernandez MJ, Alvarado-Martel D, Miranda-Calderin G, Ugarte-Lopetegui A, Gonzalez-Medina RA, et al. Prehabilitation for bariatric surgery: a randomized, controlled trial protocol and pilot study. 2021;13. doi:10.3390/nu13092903 | Wrong intervention |
| 90 | [151] | 2021 | Pierro L, Coluzzi I, Paone E, Scappaticci L, Capoccia D, Iossa A, et al. Telematics pre-operative psychological and nutritional assessment in candidates for bariatric surgery during COVID-19 phase 2: a pilot prospective observational study. Minerva Surgery. 2021;76: 57–61. | Wrong intervention |
| 91 | [152] | 2021 | Dos Santos EM, de Lima DSC, Padilha BM, Cabral PC, do Nascimento LCP, Feitosa PHF, et al. Vitamin D in the preoperative and postoperative periods of bariatric surgery. Obes Surg. 2021;31: 2723–2728. | Wrong intervention |
| 92 | [153] | 2021 | Martin-Fernandez KW, Marek RJ, Heinberg LJ, Ben-Porath YS. Six-year bariatric surgery outcomes: the predictive and incremental validity of presurgical psychological testing. Surgery for Obesity and Related Diseases. 2021;17: 1008–1016. | Wrong outcome |
| 93 | [154] | 2021 | Garcia-Delgado Y, Lopez-Madrazo-hernandez MJ, Alvarado-Martel D, Miranda-Calderin G, Ugarte-Lopetegui A, Gonzalez-Medina RA, et al. Prehabilitation for bariatric surgery: a randomized, controlled trial protocol and pilot study. 2021;13. doi:10.3390/nu13092903 | Wrong outcome |
| 94 | [155] | 2021 | de Las Heras SGG, Fernández CG, Tovar JR, Fernández-Aceñero MJ. Preoperative management of obese patients undergoing bariatric surgery: role of endoscopy and Helicobacter eradication. Obes Res Clin Pract. 2021;15: 289–290. | Wrong outcome |
| 95 | [156] | 2021 | NCT04962776. Preoperative Coadministration of Low-concentration Carbohydrate and Nitrates Loading vs Low- Concentration Carbohydrate Loading Alone in Patients Undergoing Open Gynecological Surgeries. 2021. Available: https://www.cochranelibrary.com/central/doi/10.1002/central/CN-02296697/full | Protocol |
| 96 | [157] | 2021 | NCT04942093. Nutritional Impact of a Hypocaloric Hyperprotein Diet Before Obesity Surgery. 2021. Available: https://www.cochranelibrary.com/central/doi/10.1002/central/CN-02289927/full | Protocol |
| 97 | [158] | 2021 | Griffin SB, Ross LJ, Burstow MJ, Desbrow B, Palmer MA. Efficacy of a dietitian-led very low calorie diet (VLCD) based model of care to facilitate weight loss for obese patients | Without comparison |

| | | | | |
|---|---|---|---|---|
| | | | prior to elective, non-bariatric surgery. J Hum Nutr Diet. 2021;34: 188–198. doi:10.1111/jhn.12819 | |
| 98 | [159] | 2021 | Aljaaly EA. Perioperative nutrition care and dietetic practices in the scope of bariatric surgery in Saudi Arabia using adapted protocols for evaluation. SAGE Open Med. 2021;9: 20503121211036136. doi:10.1177/20503121211036136 | Without comparison |
| 99 | [160] | 2021 | Romeijn MM, Kolen AM, Holthuijsen DDB, Janssen L, Schep G, Leclercq WKG, et al. Effectiveness of a Low-Calorie Diet for Liver Volume Reduction Prior to Bariatric Surgery: a Systematic Review. Obes Surg. 2021;31: 350–356. doi:10.1007/s11695-020-05070-6 | Without comparison |
| 100 | [161] | 2021 | Kindel TL. Comment on: Relationship between bariatric surgery outcomes and the preoperative gastrointestinal microbiota: a cohort study. Surgery for Obesity and Related Diseases. 2021;17: 899–900. | Wrong type publication |
| 101 | [162] | 2021 | Bellicha A, A van BM, Battista F, Beaulieu K, Blundell JE, Busetto L, et al. Effect of exercise training before and after bariatric surgery: A systematic review and meta-analysis. Obes Rev. 2021;22: e13296. doi:10.1111/obr.13296 | Wrong type publication |
| 102 | [163] | 2021 | Romeijn MM, Kolen AM, Holthuijsen DDB, Janssen L, Schep G, Leclercq WKG, et al. Effectiveness of a Low-Calorie Diet for Liver Volume Reduction Prior to Bariatric Surgery: a Systematic Review. Obes Surg. 2021;31: 350–356. doi:10.1007/s11695-020-05070-6 | Wrong type publication |
| 103 | [164] | 2021 | Jeram M, Mohamed F, Coomarasamy C, MacCormick AD. Are There Ethnic Differences in Micronutrient Deficiencies in Preoperative Bariatric Patients? A Systematic Review and Meta-analysis. Obes Surg. 2021;31: 5005–5021. doi:10.1007/s11695-021-05625-1 | Wrong type publication |
| 104 | [165] | 2021 | Griffin SB, Palmer MA, Strodl E, Lai R, Burstow MJ, Ross LJ. Elective Surgery in Adult Patients with Excess Weight: Can Preoperative Dietary Interventions Improve Surgical Outcomes? A Systematic Review. Nutrients. 2021;13. doi:10.3390/nu13113775 | Wrong type publication |
| 105 | [166] | 2021 | Pedro A. Preoperative Risk Factors for Early Postoperative Bleeding after Roux-en-Y Gastric Bypass: A Systematic Review and Meta-Analysis. 2021. | Wrong type publication |
| 106 | [167] | 2021 | Pavlovic N, Boland RA, Brady B, Genel F, Harris IA, Flood VM, et al. Effect of weight-loss diets prior to elective surgery on postoperative outcomes in obesity: A systematic review and meta-analysis. Clin Obes. 2021;11: e12485. doi:10.1111/cob.12485 | Wrong type publication |
| 107 | [168] | 2021 | Ha J, Kwon Y, Kwon JW, Kim D, Park SH, Hwang J, et al. Micronutrient status in bariatric surgery patients receiving postoperative supplementation per guidelines: Insights from a systematic review and meta-analysis of longitudinal studies. Obes Rev. 2021;22: e13249. doi:10.1111/obr.13249 | Wrong type publication |
| 108 | [169] | 2021 | Alimi Y, Azagury DE. Comment on: Preoperative weight loss is linked to improved mortality and leaks following elective bariatric surgery: an analysis of 548,597 patients from 2015 to 2018. Surgery for Obesity and Related Diseases. 2021;17: e59–e60. | Wrong type publication |
| 109 | [170] | 2021 | Gandotra C, Basam M, Mahajan A, Ngwa J, Ortega G, Tran D, et al. Characteristics and resolution of hypertension in obese African American bariatric cohort. Sci Rep. 2021;11: 1683. doi:10.1038/s41598-021-81360-y | Wrong type publication |
| 110 | [171] | 2021 | Stefura T, Zapała B, Gosiewski T, Krzysztofik M, Skomarovska O, Major P. Relationship between bariatric surgery outcomes and the preoperative gastrointestinal microbiota: a cohort study. Surgery for Obesity and Related Diseases. 2021;17: 889–899. | Wrong type publication |
| 111 | [172] | 2021 | Yang Y, Niu L, Mu L, Wu R, Yang L, Tong S, et al. The Remission of Type 2 Diabetes Mellitus in Chinese Patients After Metabolic Surgery and Its Preoperative Contributing Factors: A Cohort Study. 2021. | Wrong type publication |
| 112 | [173] | 2021 | Skogar ML, Sundbom M. Preoperative chronic opioid use and its impact on early complications in bariatric surgery: a Swedish nationwide cohort study of 56,183 patients. Surg Obes Relat Dis. 2021;17: 1256–1262. doi:10.1016/j.soard.2021.04.008 | Wrong type publication |
| 113 | [174] | 2021 | Zanley E, Shah ND, Craig C, Lau JN, Rivas H, McLaughlin T. Guidelines for gastrostomy tube placement and enteral nutrition in patients with severe, refractory hypoglycemia after gastric bypass. Surg Obes Relat Dis. 2021;17: 456–465. doi:10.1016/j.soard.2020.09.026 | Wrong type publication |
| 114 | [175] | 2020 | Ozeki KA, Tran SA, Cheung R, Eisenberg D. Preoperative endoscopic findings in veterans undergoing bariatric surgery: prevalence and predictors of Barrett's esophagus. Obes Surg. 2020;30: 657–663. | Wrong population |

| | | | | |
|---|---|---|---|---|
| 115 | [176] | 2020 | Joshi M, Lieb D, Gresens A, Jenkins E. Are SGLT-2 inhibitors safe in patients undergoing bariatric surgery? Endocrine Practice. 2020;26: 118–119. Available: http://www.epistemonikos.org/documents/e7305b25d1575e0dd610a4f9d804ee4e1d3eab61 | Wrong intervention |
| 116 | [177] | 2020 | Sakran N, Dar R, Assalia A, Neeman Z, Farraj M, Sherf-Dagan S, et al. The use of Ursolit for gallstone prophylaxis following bariatric surgery: a randomized-controlled trial. 2020;72: 1125-1133. doi:10.1007/s13304-020-00850-2 | Wrong intervention |
| 117 | [178] | 2020 | Muñoz-Rodríguez JR, Rodríguez-Cano T, Polo F, Sáenz-Mateos L, Agarrado A, Segura E, et al. The neuroendocrine and metabolic outcomes of bariatric surgery depend on presurgical control over eating. Neuroendocrinology. 2020;110: 63–69. | Wrong intervention |
| 118 | [179] | 2020 | Sheldon RR, Holtestaul TA, Morte DR, Jones IF, Forte DM, Bingham JR. Influence of preoperative psychotropic medications on opioid requirements and outcomes following bariatric surgery. The American Journal of Surgery. 2020;219: 841–845. | Wrong outcome |
| 119 | [180] | 2020 | Muñoz-Rodríguez JR, Rodríguez-Cano T, Polo F, Sáenz-Mateos L, Agarrado A, Segura E, et al. The neuroendocrine and metabolic outcomes of bariatric surgery depend on presurgical control over eating. Neuroendocrinology. 2020;110: 63–69. | Wrong outcome |
| 120 | [181] | 2020 | Muñoz-Rodríguez JR, Rodríguez-Cano T, Polo F, Sáenz-Mateos L, Agarrado A, Segura E, et al. The neuroendocrine and metabolic outcomes of bariatric surgery depend on presurgical control over eating. Neuroendocrinology. 2020;110: 63–69. | Wrong outcome |
| 121 | [182] | 2020 | Pouwels S, Sanches EE, Cagiltay E, Severin R, Philips SA. Perioperative Exercise Therapy in Bariatric Surgery: Improving Patient Outcomes. Diabetes Metab Syndr Obes. 2020;13: 1813–1823. doi:10.2147/DMSO.S215157 | Wrong outcome |
| 122 | [183] | 2020 | Moon RC, Brazzi-Smith G, Teixeira A, Jawad M. Preoperative Exposure to Low-Calorie Sweeteners and Bariatric Surgery Outcomes. Obes Surg. 2020;30: 5012–5019. doi:10.1007/s11695-020-04931-4 | Wrong outcome |
| 123 | [184] | 2020 | Tan SYT, Loi PL, Lim CH, Ganguly S, Syn N, Tham KW, et al. Preoperative Weight Loss via Very Low Caloric Diet (VLCD) and Its Effect on Outcomes After Bariatric Surgery. Obes Surg. 2020;30: 2099–2107. doi:10.1007/s11695-020-04446-y | Without comparison |
| 124 | [185] | 2020 | Sivakumar J, Chong L, Ward S, Sutherland TR, Read M, Hii MW. Body Composition Changes Following a Very-Low-Calorie Pre-Operative Diet in Patients Undergoing Bariatric Surgery. Obes Surg. 2020;30: 119–126. doi:10.1007/s11695-019-04174-y | Without comparison |
| 125 | [186] | 2020 | Wallenius V, Elias E, Elebring E, Haisma B, Casselbrant A, Larraufie P, et al. Suppression of enteroendocrine cell glucagon-like peptide (GLP)-1 release by fat-induced small intestinal ketogenesis: a mechanism targeted by Roux-en-Y gastric bypass surgery but not by preoperative very-low-calorie diet. Gut. 2020;69: 1423–1431. doi:10.1136/gutjnl-2019-319372 | Without comparison |
| 126 | [187] | 2020 | Pouwels S, Sanches EE, Cagiltay E, Severin R, Philips SA. Perioperative Exercise Therapy in Bariatric Surgery: Improving Patient Outcomes. Diabetes Metab Syndr Obes. 2020;13: 1813–1823. doi:10.2147/DMSO.S215157 | Without comparison |
| 127 | [188] | 2020 | Bettini S, Belligoli A, Fabris R, Busetto L. Diet approach before and after bariatric surgery. Rev Endocr Metab Disord. 2020;21: 297–306. doi:10.1007/s11154-020-09571-8 | Without comparison |
| 128 | [189] | 2020 | Pinto SL, Juvanhol LL, A da S, Shivappa N, Hébert JR, Bressan J. The Preoperative Dietary Inflammatory Index Predicts Changes in Cardiometabolic Risk Factors After 12 Months of Roux-en-Y Gastric Bypass. Obes Surg. 2020;30: 3932–3939. doi:10.1007/s11695-020-04756-1 | Without comparison |
| 129 | [190] | 2020 | Tan SYT, Loi PL, Lim CH, Ganguly S, Syn N, Tham KW, et al. Preoperative Weight Loss via Very Low Caloric Diet (VLCD) and Its Effect on Outcomes After Bariatric Surgery. Obes Surg. 2020;30: 2099–2107. doi:10.1007/s11695-020-04446-y | Without comparison |
| 130 | [191] | 2020 | El Ansari W, El-Menyar A, Sathian B, Al-Thani H, Al-Kuwari M, Al-Ansari A. Is routine preoperative esophagogastroduodenoscopy prior to bariatric surgery mandatory? Systematic review and meta-analysis of 10,685 patients. Obes Surg. 2020;30: 3073–3083. | Wrong type publication |
| 131 | [192] | 2020 | El Ansari W, El-Menyar A. Is routine preoperative esophagogastroduodenscopy prior to bariatric surgery mandatory? protocol for a systematic review and meta-analysis. Int J Surg Protoc. 2020;22: 1–5. | Wrong type publication |
| 132 | [193] | 2020 | Evans G, Barker A, Simon L, Kushnir V. High cost for low yield: a systematic review and meta-analysis to assess cost of routine preoperative esophagogastroduodenoscopy before bariatric surgery. J Clin Gastroenterol. 2020;54: 398–404. | Wrong type publication |

| | | | | |
|---|---|---|---|---|
| 133 | [194] | 2020 | Qiang H, Yuanshui S. Comment on "preoperative administration of Omega-3 fatty acids on postoperative pain and acute-phase reactants in patients undergoing Roux-en-Y gastric bypass: a randomized clinical trial." 2020;39: 1301. doi:10.1016/j.clnu.2020.02.028 | Wrong type publication |
| 134 | [195] | 2020 | Abbott S, Lawson J, Singhal R, Parretti HM, Tahrani AA. Weight loss during medical weight management does not predict weight loss after bariatric surgery: a retrospective cohort study. Surg Obes Relat Dis. 2020;16: 1723–1730. doi:10.1016/j.soard.2020.06.049 | Wrong type publication |
| 135 | [196] | 2020 | AlEid A, Al Balkhi A, Hummedi A, Alshaya A, Abukhater M, Al Mtawa A, et al. The utility of esophagogastroduodenoscopy and Helicobacter pylori screening in the preoperative assessment of patients undergoing bariatric surgery: A cross-sectional, single-center study in Saudi Arabia. Saudi Journal of Gastroenterology. 2020;26: 32–38. | Wrong type publication |
| 136 | [197] | 2020 | Raatz SK, Johnson LK, Caliquary A, King WC, Kalarchian MA, Devlin MJ, et al. Reported nutrient intake over 7 years after Roux-en-Y gastric bypass in the Longitudinal Assessment of Bariatric Surgery-3 (LABS-3) psychosocial study. Surg Obes Relat Dis. 2020;16: 1022–1029. doi:10.1016/j.soard.2020.04.007 | Wrong type publication |
| 137 | [198] | 2020 | Galtier F, Pattou F, Czernichow S, Disse E, Ritz P, Chevallier JM, et al. Bariatric surgery and the perioperative management of type 2 diabetes: Practical guidelines. J Visc Surg. 2020;157: 13–21. doi:10.1016/j.jviscsurg.2019.07.012 | Wrong type publication |
| 138 | [199] | 2020 | O'Kane M, Parretti HM, Pinkney J, Welbourn R, Hughes CA, Mok J, et al. British Obesity and Metabolic Surgery Society Guidelines on perioperative and postoperative biochemical monitoring and micronutrient replacement for patients undergoing bariatric surgery-2020 update. Obes Rev. 2020;21: e13087. doi:10.1111/obr.13087 | Wrong type publication |
| 139 | [200] | 2019 | Kim Y, Sysko R, Michaeledes A, Ramos T, Hildebrandt T. Effects of smartphone coaching intervention on dietary intake for bariatric surgery candidates: a pilot randomized controlled trial. 2019;24: 220-221. doi:10.1017/S1092852919000014 | Wrong intervention |
| 140 | [201] | 2019 | Gonzalez L, Soto J, Leyton B, Cancino J, Olivares M, Pino J, et al. Isocaloric high intensity interval training and continuous training on body composition and fitness in bariatric surgery candidates with morbid obesity. 2019;29: 607. doi:10.1007/s11695-019-04101-1 | Wrong intervention |
| 141 | [202] | 2019 | Saurabh S, Gao Y, Maduka S, Smith L, Lasley R, Singh N. Is Transdermal Multivitamin Patch Effective in Gastric Bypass Patients? Obes Surg. 2019;29: 3818–3823. doi:10.1007/s11695-019-04070-5 | Wrong intervention |
| 142 | [203] | 2019 | Martin-Fernandez KW, Heinberg LJ, Ben-Porath YS. Using the preoperative psychological evaluation to determine psychosocial risk factors for CPAP nonadherence among bariatric surgery candidates. Surg Obes Relat Dis. 2019;15: 2115–2120. doi:10.1016/j.soard.2019.07.012 | Wrong outcome |
| 143 | [204] | 2019 | Husain TM, Salgado CJ, Mundra LS, Perez C, AlQattan HT, Bustillo E, et al. Abdominal Etching: Surgical Technique and Outcomes. Plast Reconstr Surg. 2019;143: 1051–1060. doi:10.1097/PRS.0000000000005486 | Wrong outcome |
| 144 | [205] | 2019 | Marshall S, Mackay H, Rich G, Isenring E. Do intensive preoperative and postoperative behavioural interventions impact on health-related bariatric surgery outcomes? A systematic review. Clinical Nutrition. 2019;38: s274. | Wrong outcome |
| 145 | [206] | 2019 | Serafim MP, Santo MA, Gadducci AV, Scabim VM, Cecconello I, de Cleva R. Very low-calorie diet in candidates for bariatric surgery: Change in body composition during rapid weight loss. Clinics. 2019;74. doi:10.6061/clinics/2019/e560 | Without comparison |
| 146 | [207] | 2019 | Kim Y, Sysko R, Michaeledes A, Ramos T, Hildebrandt T. Effects of smartphone coaching intervention on dietary intake for bariatric surgery candidates: a pilot randomized controlled trial. 2019;24: 220-221. doi:10.1017/S1092852919000014 | Without comparison |
| 147 | [208] | 2019 | Elrefai M. Value of preoperative diet before sleeve gastrectomy: a prospective randomized study. 2019;29: 860. doi:10.1007/s11695-019-04101-1 | Without comparison |
| 148 | [209] | 2019 | Serafim MP, Santo MA, Gadducci A V, Scabim VM, Cecconello I, R de C. Very low-calorie diet in candidates for bariatric surgery: change in body composition during rapid weight loss. Clinics (Sao Paulo). 2019;74: e560. doi:10.6061/clinics/2019/e560 | Without comparison |
| 149 | [210] | 2019 | Marshall S, Mackay H, Rich G, Isenring E. Do intensive preoperative and postoperative behavioural interventions impact on health-related bariatric surgery outcomes? A systematic review. Clinical Nutrition. 2019;38: s274. | Wrong type publication |

| | | | | |
|---|---|---|---|---|
| 150 | [211] | 2019 | Lee Y, Dang JT, Switzer N, Malhan R, Birch DW, Karmali S. Bridging interventions before bariatric surgery in patients with BMI ≥ 50 kg/m(2): a systematic review and meta-analysis. Surg Endosc. 2019;33: 3578–3588. doi:10.1007/s00464-019-07027-y | Wrong type publication |
| 151 | [212] | 2019 | Mackey ER, Jacobs M, Nadler EP. Preoperative exercise as a predictor of weight loss in adolescents and young adults following sleeve gastrectomy: a cohort study. Surg Obes Relat Dis. 2019;15: 1051–1057. doi:10.1016/j.soard.2019.03.039 | Wrong type publication |
| 152 | [213] | 2019 | Abebe BT, Weiss M, Modess C, Roustom T, Tadken T, Wegner D, et al. Effects of the P-Glycoprotein Inhibitor Clarithromycin on the Pharmacokinetics of Intravenous and Oral Trospium Chloride: a 4-Way Crossover Drug-Drug Interaction Study in Healthy Subjects. 2019;59: 1319-1330. doi:10.1002/jcph.1421 | Wrong type publication |
| 153 | [214] | 2019 | Shoar S, Naini AA, Aleassa EM, Naderan M, Mahmoodzadeh H. Letter to Editor regarding "preoperative liver shrinking diet for bariatric surgery may impact wound healing: a randomized controlled trial." 2019;15: 669-670. doi:10.1016/j.soard.2018.10.018 | Wrong type publication |
| 154 | [215] | 2019 | Hubert PA, Papasavas P, Stone A, Swede H, Huedo-Medina TB, Tishler D, et al. Associations between Weight Loss, Food Likes, Dietary Behaviors, and Chemosensory Function in Bariatric Surgery: A Case-Control Analysis in Women. Nutrients. 2019;11. doi:10.3390/nu11040804 | Wrong type publication |
| 155 | [216] | 2019 | Xiaosong W, Chongyu S, Xuqi S, Peiwu Y, Yongliang Z. Risk Factors for Relapse of Hyperglycemia after Laparoscopic Roux-en-Y Gastric Bypass in T2DM Obese Patients: a 5-Year Follow-Up of 24 Cases. Obes Surg. 2019;29: 1164–1168. doi:10.1007/s11695-018-03656-9 | Wrong type publication |
| 156 | [217] | 2018 | Tewksbury CM. Preoperative patient contacts and postoperative weight loss after bariatric surgery: a retrospective study. 2018. | Wrong population |
| 157 | [218] | 2018 | Tang T, Abbott S, W le RC, Wilson V, Singhal R, Bellary S, et al. Preoperative weight loss with glucagon-like peptide-1 receptor agonist treatment predicts greater weight loss achieved by the combination of medical weight management and bariatric surgery in patients with type 2 diabetes: A longitudinal analysis. Diabetes Obes Metab. 2018;20: 745–748. doi:10.1111/dom.13131 | Wrong intervention |
| 158 | [219] | 2018 | Lemanu DP, Singh PP, Shao RY, Pollock TT, MacCormick AD, Arroll B, et al. Text messaging improves preoperative exercise in patients undergoing bariatric surgery. ANZ J Surg. 2018. doi:10.1111/ans.14418 | |
| 159 | [220] | 2018 | Morton J, Rivas H, Garcia L, Azagury DE. Utilizing Low-Dose Phentermine for Preoperative Weight Loss Prior to Bariatric Surgery: A Prospective, Randomized, and Placebo-Controlled Trial. Surgery for Obesity and Related Diseases. 2018;14: S33. | Wrong intervention |
| 160 | [221] | 2018 | Taguchi M, Weiss AL. Examining pre-operative comorbidities and weight loss in adolescent bariatric surgery. Journal of Adolescent Health. 2018;62: S124. | Wrong intervention |
| 161 | [222] | 2018 | Tang T, Abbott S, W le RC, Wilson V, Singhal R, Bellary S, et al. Preoperative weight loss with glucagon-like peptide-1 receptor agonist treatment predicts greater weight loss achieved by the combination of medical weight management and bariatric surgery in patients with type 2 diabetes: A longitudinal analysis. Diabetes Obes Metab. 2018;20: 745–748. doi:10.1111/dom.13131 | Wrong intervention |
| 162 | [223] | 2018 | Lemanu DP, Singh PP, Shao RY, Pollock TT, MacCormick AD, Arroll B, et al. Text messaging improves preoperative exercise in patients undergoing bariatric surgery. ANZ J Surg. 2018. doi:10.1111/ans.14418 | Wrong intervention |
| 163 | [224] | 2018 | Morton J, Rivas H, Garcia L, Azagury DE. Utilizing Low-Dose Phentermine for Preoperative Weight Loss Prior to Bariatric Surgery: A Prospective, Randomized, and Placebo-Controlled Trial. Surgery for Obesity and Related Diseases. 2018;14: S33. | Wrong outcome |
| 164 | [225] | 2018 | Schiavo L, Pilone V, Rossetti G, Barbarisi A, Cesaretti M, Iannelli A. A 4-Week Preoperative Ketogenic Micronutrient-Enriched Diet Is Effective in Reducing Body Weight, Left Hepatic Lobe Volume, and Micronutrient Deficiencies in Patients Undergoing Bariatric Surgery: a Prospective Pilot Study. Obes Surg. 2018;28: 2215–2224. doi:10.1007/s11695-018-3145-8 | Without comparison |
| 165 | [226] | 2018 | Pilone V, Tramontano S, Renzulli M, Romano M, Cobellis L, Berselli T, et al. Metabolic effects, safety, and acceptability of very low-calorie ketogenic dietetic scheme on candidates for bariatric surgery. Surgery for Obesity and Related Diseases. 2018;14: 1013–1019. doi:10.1016/j.soard.2018.03.018 | Without comparison |

| | | | | |
|---|---|---|---|---|
| 166 | [227] | 2018 | Pilone V, Tramontano S, Renzulli M, Romano M, Cobellis L, Berselli T, et al. Metabolic effects, safety, and acceptability of very low-calorie ketogenic dietetic scheme on candidates for bariatric surgery. Surg Obes Relat Dis. 2018;14: 1013–1019. doi:10.1016/j.soard.2018.03.018 | Without comparison |
| 167 | [228] | 2018 | Funes DR, Menzo E Lo, Ganga RR, Szomstein S, Rosenthal RJ. Is there a Correlation Between Length of Preoperative Diet and Weight loss After Bariatric Surgery? Surgery for Obesity and Related Diseases. 2018;14: S116. | Without comparison |
| 168 | [229] | 2018 | Kraus R, Stekhoven DJ, Leupold U, Marti WR. Linear Mixed Effects Analysis Reveals the Significant Impact of Preoperative Diet Success on Postoperative Weight Loss in Gastric Bypass Surgery. Obes Surg. 2018;28: 2473–2480. doi:10.1007/s11695-018-3189-9 | Without comparison |
| 169 | [230] | 2018 | Guerrero Perez F, Sánchez-González J, Sánchez I, Jiménez-Murcia S, Granero R, Simó-Servat A, et al. Food addiction and preoperative weight loss achievement in patients seeking bariatric surgery. European Eating Disorders Review. 2018;26: 645–656. | Wrong type publication |
| 170 | [231] | 2018 | Naseer F, Shabbir A, Livingstone B, Price R, Syn NL, Flannery O. The Efficacy of Energy-Restricted Diets in Achieving Preoperative Weight Loss for Bariatric Patients: a Systematic Review. Obes Surg. 2018;28: 3678–3690. doi:10.1007/s11695-018-3451-1 | Wrong type publication |
| 171 | [232] | 2018 | Holderbaum M, Casagrande DS, Sussenbach S, Buss C. Effects of very low calorie diets on liver size and weight loss in the preoperative period of bariatric surgery: a systematic review. Surgery for Obesity and Related Diseases. 2018;14: 237–244. | Wrong type publication |
| 172 | [233] | 2018 | Neovius M, Bruze G, Jacobson P, Sjöholm K, Johansson K, Granath F, et al. Risk of suicide and non-fatal self-harm after bariatric surgery: results from two matched cohort studies. Lancet Diabetes Endocrinol. 2018;6: 197–207. doi:10.1016/S2213-8587(17)30437-0 | Wrong type publication |
| 173 | [234] | 2018 | Jassil FC, Carnemolla A, Kingett H, Paton B, O'Keeffe AG, Doyle J, et al. Protocol for a 1-year prospective, longitudinal cohort study of patients undergoing Roux-en-Y gastric bypass and sleeve gastrectomy: the BARI-LIFESTYLE observational study. BMJ Open. 2018;8: e020659. doi:10.1136/bmjopen-2017-020659 | Wrong type publication |
| 174 | [235] | 2018 | Boshier PR, Fehervari M, Markar SR, Purkayastha S, Spanel P, Smith D, et al. Variation in Exhaled Acetone and Other Ketones in Patients Undergoing Bariatric Surgery: a Prospective Cross-sectional Study. Obes Surg. 2018;28: 2439–2446. doi:10.1007/s11695-018-3180-5 | Wrong type publication |
| 175 | [236] | 2018 | Goldstein SP, Thomas JG, Vithiananthan S, Blackburn GA, Jones DB, Webster J, et al. Multi-sensor ecological momentary assessment of behavioral and psychosocial predictors of weight loss following bariatric surgery: study protocol for a multicenter prospective longitudinal evaluation. BMC Obes. 2018;5: 27. doi:10.1186/s40608-018-0204-6 | Wrong type publication |
| 176 | [237] | 2018 | Moon RC, Ghanem M, Teixeira AF, N DLC-M, Young MK, Domkowski P, et al. Assessing risk factors, presentation, and management of portomesenteric vein thrombosis after sleeve gastrectomy: a multicenter case-control study. Surg Obes Relat Dis. 2018;14: 478–483. doi:10.1016/j.soard.2017.10.013 | Wrong type publication |
| 177 | [238] | 2017 | Baltieri L. Efeitos da perda de peso pré-operatória e da cirurgia bariátrica na função pulmonar e no perfil inflamatório sistêmico e pulmonar de obesos asmáticos= Effects of preoperative weight loss and bariatric surgery in pulmonary function and systemic and pulmonary inflammatory profile of asthmatics obese. 2017. | Other language |
| 178 | [239] | 2017 | Pineda O, Maydón HG, Amado M, Sepúlveda EM, Guilbert L, Espinosa O, et al. A prospective study of the conservative management of asymptomatic preoperative and postoperative gallbladder disease in bariatric surgery. Obes Surg. 2017;27: 148–153. | Wrong population |
| 179 | [240] | 2017 | Morton J, Khoury H, Azagury D, Rivas H. Early experience with low-dose phentermine for preoperative bariatric weight loss: a prospective randomized trial. 2017;13: S63. | Wrong intervention |
| 180 | [241] | 2017 | Shah R, Shah S, Sawant A, Shah P, Gangwani J, Khamkar A, et al. Can pharmacotherapy be superior to diet for preoperative bariatric surgery preparation? Pre-operative management. 2017;27: 137. doi:10.1007/s11695-017-2774-7 | Wrong intervention |
| 181 | [242] | 2017 | Raftopoulos I, Chiapperino M, Michelakis M, Davidson E. Successful preoperative lifestyle intervention is associated with a greater% total body weight loss and BMI reduction at 12 months after bariatric surgery. Surgery for Obesity and Related Diseases. 2017;13: S146–S147. | Wrong intervention |

| | | | | |
|---|---|---|---|---|
| 182 | [243] | 2017 | Watanabe A, Seki Y, Haruta H, Kikkawa E, Kasama K. Preoperative Weight Loss and Operative Outcome After Laparoscopic Sleeve Gastrectomy. Obes Surg. 2017;27: 2515–2521. doi:10.1007/s11695-017-2697-3 | Wrong outcome |
| 183 | [244] | 2017 | Aelfers SCW, Schijns W, Ploeger N, Janssen IMC, Berends FJ, Aarts EO. Patients' preoperative estimate of target weight and actual outcome after bariatric surgery. Obes Surg. 2017;27: 1729–1734. | Wrong outcome |
| 184 | [245] | 2017 | Taha O, Abdelaal M, Abozeid M, Askalany A, Alaa M. Outcomes of One Anastomosis Gastric Bypass in 472 Diabetic Patients. Obes Surg. 2017;27: 2802–2810. doi:10.1007/s11695-017-2711-9 | Wrong outcome |
| 185 | [246] | 2017 | King WC, Chen JY, Belle SH, Courcoulas AP, Dakin GF, Flum DR, et al. Use of prescribed opioids before and after bariatric surgery: prospective evidence from a U.S. multicenter cohort study. Surg Obes Relat Dis. 2017;13: 1337–1346. doi:10.1016/j.soard.2017.04.003 | Wrong outcome |
| 186 | [247] | 2017 | Sherf Dagan S, Keidar A, Raziel A, Sakran N, Goitein D, Shibolet O, et al. Do Bariatric Patients Follow Dietary and Lifestyle Recommendations during the First Postoperative Year? 2017;27: 2258-2271. doi:10.1007/s11695-017-2633-6 | Without comparison |
| 187 | [248] | 2017 | Elrefai M. Value of low calorie diet before sleeve gastrectomy: prospectieve randomised study. pre-operative management. 2017;27: 813. doi:10.1007/s11695-017-2774-7 | Without comparison |
| 188 | [249] | 2017 | King WC, Chen JY, Belle SH, Courcoulas AP, Dakin GF, Flum DR, et al. Use of prescribed opioids before and after bariatric surgery: prospective evidence from a U.S. multicenter cohort study. Surg Obes Relat Dis. 2017;13: 1337–1346. doi:10.1016/j.soard.2017.04.003 | Wrong type publication |
| 189 | [250] | 2017 | Motamedi MAK, Barzin M, Ebrahimi M, Ebrahimi R, Khalaj A. Severe fatal protein malnutrition and liver failure in a morbidly obese patient after mini-gastric bypass surgery: Case report. Int J Surg Case Rep. 2017;33: 71–74. doi:10.1016/j.ijscr.2017.02.033 | Wrong type publication |
| 190 | [251] | 2017 | Ip WTK, Chandramouli C, Smith JA, McLennan PL, Pepe S, Delbridge LMD. A Small Cohort Omega-3 PUFA Supplement Study: implications of Stratifying According to Lipid Membrane Incorporation in Cardiac Surgical Patients. 2017;26: 846-855. doi:10.1016/j.hlc.2016.12.007 | Wrong type publication |
| 191 | [252] | 2017 | de Souza MC, dos Santos SR, de Fontes Pereira WD, de Arruda AJCG, da Costa Santos IB, dos Santos Oliveira J, et al. Nursing Assistance to the Patient in the Preoperative and Postoperative of Bariatric Surgery. | Wrong intervention |
| 192 | [253] | 2016 | Mensorio M, Costa-Júnior Á. Intervención psicológica a candidatos de cirugía bariátrica en un hospital público de Brasil. Rev peru med exp salud publica. 2016;33: 120–127. Available: http://www.rpmesp.ins.gob.pe/index.php/rpmesp/article/view/1941/1850 | Other language |
| 193 | [254] | 2016 | Lee W-J, Chong K, Chen S-C, Zachariah J, Ser K-H, Lee Y-C, et al. Preoperative prediction of type 2 diabetes remission after gastric bypass surgery: a comparison of DiaRem scores and ABCD scores. Obes Surg. 2016;26: 2418–2424. | Wrong population |
| 194 | [255] | 2016 | Guisado-Macías JA, Méndez-Sánchez F, Baltasar-Tello I, Zamora-Rodríguez FJ, Escudero-Sánchez AB, Vaz-Leal FJ. Fluoxetine, topiramate, and combination of both to stabilize eating behavior before bariatric surgery. Actas Esp Psiquiatr. 2016;44: 93–6. Available: https://pubmed.ncbi.nlm.nih.gov/27254401/ | Wrong intervention |
| 195 | [256] | 2016 | Delgado Floody P, Caamaño Navarrete F, Osorio Poblete A, Jerez Mayorga D. Variaciones en el estado nutricional, presión arterial y capacidad cardiorrespiratoria de obesos candidatos a cirugía bariátrica: beneficios del ejercicio físico con apoyo multidisciplinar. Nutr hosp. 2016;33: 54–58. doi:10.20960/nh.v33i1.16 | Wrong intervention |
| 196 | [257] | 2016 | Siddiq G, Aziz W, Pervez MB, Haider MI, Hussain S V, Khan N. Early Laparoscopic Sleeve Gastrectomy Outcomes in Terms of Weight Loss. J Coll Physicians Surg Pak. 2016;26: 169–72. Available: https://pubmed.ncbi.nlm.nih.gov/26975944/ | Wrong outcome |
| 197 | [258] | 2016 | Hong J, Park S. Preoperative Nutritional Management of Patients with Morbid Obesity. Journal of Metabolic and Bariatric Surgery. 2016; 53–61. Available: http://dx.doi.org/ | Wrong outcome |
| 198 | [259] | 2016 | Nielsen L V., Nielsen MS, Schmidt JB, Pedersen SD, Sjödin A. Efficacy of a liquid low-energy formula diet in achieving preoperative target weight loss before bariatric surgery. J Nutr Sci. 2016;5. doi:10.1017/jns.2016.13 | Without comparison |
| 199 | [260] | 2016 | Ross LJ, Wallin S, Osland EJ, Memon MA. Commercial Very Low Energy Meal Replacements for Preoperative Weight Loss in Obese Patients: a Systematic Review. Obes Surg. 2016;26: 1343–51. doi:10.1007/s11695-016-2167-3 | Without comparison |

| | | | | |
|---|---|---|---|---|
| 200 | [261] | 2016 | Appel JE, Ift F, Kißler H, Kloos C, Lehmann T, Strauß B, et al. Influence of Attachment Style on the Outcome of Bariatric Surgery - A Pilot Study. 2016;66: 465-472. doi:10.1055/s-0042-118191 | Without comparison |
| 201 | [262] | 2016 | Á BRM, D MR, J OS, L SSJ. Nutritional alterations after very low-calorie diet before bariatric surgery. Cir Esp. 2016;94: 159–64. doi:10.1016/j.ciresp.2015.06.003 | Without comparison |
| 202 | [263] | 2016 | Keith C, Goss L, Blackledge C, Stahl R, Grams J. Insurance-mandated pre-operative diet and outcomes following bariatric surgery. Surgery for Obesity and Related Diseases. 2016;12: S31–S32. | Without comparison |
| 203 | [264] | 2016 | Cleveland E, Peirce G, Brown S, Freemyer J, Rice W, Lee L, et al. A short-duration restrictive diet reduces visceral adiposity in the morbidly obese surgical patient. Am J Surg. 2016;212: 927–930. doi:10.1016/j.amjsurg.2016.01.040 | Without comparison |
| 204 | [265] | 2016 | Ross LJ, Wallin S, Osland EJ, Memon MA. Commercial Very Low Energy Meal Replacements for Preoperative Weight Loss in Obese Patients: a Systematic Review. Obes Surg. 2016;26: 1343–51. doi:10.1007/s11695-016-2167-3 | Wrong type publication |
| 205 | [266] | 2016 | Stewart F, Avenell A. Behavioural Interventions for Severe Obesity Before and/or After Bariatric Surgery: a Systematic Review and Meta-analysis. Obes Surg. 2016;26: 1203–14. doi:10.1007/s11695-015-1873-6 | Wrong type publication |
| 206 | [267] | 2016 | Bennett S, Gostimir M, Shorr R, Mallick R, Mamazza J, Neville A. The role of routine preoperative upper endoscopy in bariatric surgery: a systematic review and meta-analysis. Surg Obes Relat Dis. 2016;12: 1116–1125. doi:10.1016/j.soard.2016.04.012 | Wrong type publication |
| 207 | [268] | 2016 | Yorke E, Dang JT, Sun WYL, Rieder S, Switzer NJ, Chow A, et al. 2016 Canadian Surgery Forum01 Intragastric balloon for management of severe obesity: a systematic review. 02 Treating type 2 diabetes with bariatric surgery—a predictive tool. 03 An update on idiopathic intracranial hypertension and bariatric surgery: a systematic review. 04 Cost-effectiveness an... Canadian Journal of Surgery. 2016;59: S77–S147. | Wrong type publication |
| 208 | [269] | 2016 | Moran J, Wilson F, Guinan E, McCormick P, Hussey J, Moriarty J. Role of cardiopulmonary exercise testing as a risk-assessment method in patients undergoing intra-abdominal surgery: a systematic review. Br J Anaesth. 2016;116: 177–91. doi:10.1093/bja/aev454 | Wrong type publication |
| 209 | [270] | 2016 | Parrott J, Frank L, Rabena R, Craggs-Dino L, Isom KA, Greiman L. American Society for Metabolic and Bariatric Surgery Integrated Health Nutritional Guidelines for the Surgical Weight Loss Patient 2016 Update: Micronutrients. Surg Obes Relat Dis. 2017;13: 727–741. doi:10.1016/j.soard.2016.12.018 | Wrong type publication |
| 210 | [271] | 2016 | Thibault R, Pichard C. Overview on nutritional issues in bariatric surgery. Curr Opin Clin Nutr Metab Care. 2016;19: 484–490. doi:10.1097/MCO.0000000000000325 | Wrong type publication |
| 211 | [272] | 2015 | Alosco ML, Spitznagel MB, Strain G, Devlin M, Cohen R, Crosby RD, et al. Pre-operative history of depression and cognitive changes in bariatric surgery patients. Psychol Health Med. 2015;20: 802–813. | Wrong population |
| 212 | [273] | 2015 | Hayes S, Napolitano MA, Lent MR, Wood GC, Gerhard GS, Irving BA, et al. The effect of insurance status on pre-and post-operative bariatric surgery outcomes. Obes Surg. 2015;25: 191–194. | Wrong population |
| 213 | [274] | 2015 | Leon S, Rouhi AD, Perez JE, Alberstadt AN, Tewksbury CM, Gershuni VM, et al. Bariatric surgery in patients with preoperative therapeutic anticoagulation: a 2015-2021 MBSAQIP database study. Surg Obes Relat Dis. 2024;20: 1260–1269. doi:10.1016/j.soard.2024.07.018 | Wrong population |
| 214 | [275] | 2015 | Courcoulas AP, Christian NJ, O'Rourke RW, Dakin G, Dellinger EP, Flum DR, et al. Preoperative factors and 3-year weight change in the Longitudinal Assessment of Bariatric Surgery (LABS) consortium. Surgery for Obesity and Related Diseases. 2015;11: 1109–1118. | Wrong intervention |
| 215 | [276] | 2015 | Wiltberger G, Bucher JN, Schmelzle M, Hoffmeister A, Dietrich A. Preoperative endoscopy and its impact on perioperative management in bariatric surgery. Dig Surg. 2015;32: 238–242. | Wrong intervention |
| 216 | [277] | 2015 | Baltieri L, Santos LA dos, Rasera-Junior I, Montebelo MI de L, Pazzianotto-Forti EM. Use of positive pressure in preoperative and intraoperative of bariatric surgery and its effect on the time of extubation. Rev Bras Anestesiol. 2015;65: 130–135. | Wrong intervention |

| | | | | |
|---|---|---|---|---|
| 217 | [278] | 2015 | Jain D, Singhal S. Endoscopic Bypass Using Endobarrier Devices: Efficacy in Treating Obesity and Metabolic Syndrome. J Clin Gastroenterol. 2015;49: 799–803. doi:10.1097/MCG.0000000000000396 | Wrong intervention |
| 218 | [279] | 2015 | Lund MT, Hansen M, Skaaby S, Dalby S, Støckel M, Floyd AK, et al. Preoperative β-cell function in patients with type 2 diabetes is important for the outcome of Roux-en-Y gastric bypass surgery. J Physiol. 2015;593: 3123–33. doi:10.1113/JP270264 | Wrong outcome |
| 219 | [280] | 2015 | NCT02418975. Pre-operative Very Low-calorie Protein-based Versus Hypocaloric Enteral Nutrition. 2015. Available: https://www.cochranelibrary.com/central/doi/10.1002/central/CN-01505802/full | Protocol |
| 220 | [281] | 2015 | Ruiz-Tovar J, Boix E, Bonete JM, Martínez R, Zubiaga L, Díez M, et al. Effect of Preoperative Eating Patterns and Preoperative Weight Loss on the Short- and Mid-term Weight Loss Results of Sleeve Gastrectomy. Cirugía Española (English Edition). 2015;93: 241–247. doi:10.1016/j.cireng.2014.04.008 | Without comparison |
| 221 | [282] | 2015 | Schiavo L, Scalera G, Sergio R, De Sena G, Pilone V, Barbarisi A. Clinical impact of Mediterranean-enriched-protein diet on liver size, visceral fat, fat mass, and fat-free mass in patients undergoing sleeve gastrectomy. Surgery for Obesity and Related Diseases. 2015;11: 1164–1170. doi:10.1016/j.soard.2015.04.003 | Without comparison |
| 222 | [283] | 2015 | Kalarchian MA, Marcus MD, Courcoulas AP, Cheng Y, Levine MD. Preoperative lifestyle intervention in bariatric surgery: a randomized clinical trial. Surgery for Obesity and Related Diseases. 2016;12: 180–187. doi:10.1016/j.soard.2015.05.004 | Without comparison |
| 223 | [284] | 2015 | Leonetti F, Campanile FC, Coccia F, Capoccia D, Alessandroni L, Puzziello A, et al. Very Low-Carbohydrate Ketogenic Diet Before Bariatric Surgery: Prospective Evaluation of a Sequential Diet. Obes Surg. 2015;25: 64–71. doi:10.1007/s11695-014-1348-1 | Without comparison |
| 224 | [285] | 2015 | Edholm D, Kullberg J, Karlsson FA, Haenni A, Ahlström H, Sundbom M. Changes in liver volume and body composition during 4 weeks of low calorie diet before laparoscopic gastric bypass. Surg Obes Relat Dis. 2015;11: 602–6. doi:10.1016/j.soard.2014.07.018 | Without comparison |
| 225 | [286] | 2015 | Marek RJ, Tarescavage AM, Ben-Porath YS, Ashton K, Rish JM, Heinberg LJ. Using presurgical psychological testing to predict 1-year appointment adherence and weight loss in bariatric surgery patients: predictive validity and methodological considerations. Surgery for Obesity and Related Diseases. 2015;11: 1171–1181. | Wrong type publication |
| 226 | [287] | 2015 | Liu RH. Do Behavioral Interventions Delivered before Bariatric Surgery Impact Weight Loss in Adults? A Systematic Scoping Review. Bariatr Surg Pract Patient Care. 2016;11: 39–48. doi:10.1089/bari.2015.0047 | Wrong type publication |
| 227 | [288] | 2015 | Chen Y, Zeng G, Tan J, Tang J, Ma J, Rao B. Impact of roux-en Y gastric bypass surgery on prognostic factors of type 2 diabetes mellitus: meta-analysis and systematic review. Diabetes Metab Res Rev. 2015;31: 653–62. doi:10.1002/dmrr.2622 | Wrong type publication |
| 228 | [289] | 2015 | Bose S, Khanna A, You J, Arora L, Qavi S, Turan A. Low serum vitamin D levels are not associated with increased postoperative pain and opioid requirements: a historical cohort study. Can J Anaesth. 2015;62: 770–6. doi:10.1007/s12630-015-0357-4 | Wrong type publication |
| 229 | [290] | 2015 | Werling M, Fändriks L, Vincent RP, Cross GF, le Roux CW, Olbers T. Erratum to "Preoperative assessment of gut hormones does not correlate to weight loss after Roux-en-Y gastric bypass surgery": Surgery for Obesity and Related Diseases 10 (2014) 822-828. Surgery for Obesity and Related Diseases. 2015;11: 1412. | Wrong type publication |
| 230 | [291] | 2015 | Parker SG, McGlone ER, Knight WR, Sufi P, Khan OA. Enoxaparin venous thromboembolism prophylaxis in bariatric surgery: A best evidence topic. Int J Surg. 2015;23: 52–6. doi:10.1016/j.ijsu.2015.09.005 | Wrong type publication |
| 231 | [292] | 2015 | Leahy CR, Luning A. Review of nutritional guidelines for patients undergoing bariatric surgery. AORN J. 2015;102: 153–60. doi:10.1016/j.aorn.2015.05.017 | Wrong type publication |
| 232 | [293] | 2014 | Gordon PC. Avaliação longitudinal psicopatológica e de personalidade de pacientes submetidos à cirurgia bariátrica: implicações prognósticas. 2014. pp. 134–134. Available: http://www.teses.usp.br/teses/disponiveis/5/5142/tde-01122014-154407/pt-br.php | Other language |
| 233 | [294] | 2014 | Chuah LL, Miras A, Olbers T, Le Roux CW. Effect of intensive preoperative and postoperative glucose management on glycaemic outcome after Roux-en-Y gastric bypass surgery. 2014;31: 94-95. doi:10.1111/dme.12378_2 | Wrong outcome |

| | | | | |
|---|---|---|---|---|
| 234 | [295] | 2014 | Nogueira I, Hrovat K. Adolescent bariatric surgery: review on nutrition considerations. Nutr Clin Pract. 2014;29: 740–6. doi:10.1177/0884533614552851 | Wrong outcome |
| 235 | [296] | 2014 | NCT02269410. Metabolic Impact of Dietary Protein Supplementation in Surgical Weight Loss. 2014. Available: https://www.cochranelibrary.com/central/doi/10.1002/central/CN-01589939/full | Protocol |
| 236 | [297] | 2014 | Baldry EL, Leeder PC, Idris IR. Pre-operative dietary restriction for patients undergoing bariatric surgery in the UK: observational study of current practice and dietary effects. Obes Surg. 2014;24: 416–21. doi:10.1007/s11695-013-1125-6 | Without comparison |
| 237 | [298] | 2014 | Els A. The preoperative use of restricted energy diets to reduce liver volume and liver fat content and improve postoperative outcome in obsese patients scheduled for bariatric surgery: a systematic review and meta-analysis. 2014. | Wrong type publication |
| 238 | [299] | 2014 | Speck RM, Bond DS, Sarwer DB, Farrar JT. A systematic review of musculoskeletal pain among bariatric surgery patients: implications for physical activity and exercise. Surg Obes Relat Dis. 2014;10: 161–70. doi:10.1016/j.soard.2013.08.001 | Wrong type publication |
| 239 | [300] | 2014 | Lefebvre P, Letois F, Sultan A, Nocca D, Mura T, Galtier F. Nutrient deficiencies in patients with obesity considering bariatric surgery: a cross-sectional study. Surg Obes Relat Dis. 2014;10: 540–6. doi:10.1016/j.soard.2013.10.003 | Wrong type publication |
| 240 | [301] | 2014 | Hyunjo KIM. Gastroplasty for Esophageal Perforation after Endoscopic Balloon Dilatation for Achalasia: Two Cases. J Korean Med Sci. 2014; 739–742. Available: http://dx.doi.org/10.3346/jkms.2014.29.5.739 | Wrong type publication |
| 241 | [302] | 2014 | Ji-Yeong AN, In CHO, CHOI Y-Y, Yoo-Min KIM, Sung-Hoon NOH. Totally Laparoscopic Roux-en-Y Gastrojejunostomy after Laparoscopic Distal Gastrectomy: Analysis of Initial 50 Consecutive Cases of Single Surgeon in Comparison with Totally Laparoscopic Billroth I Reconstruction. Yonsei med j. 2014; 162–169. Available: http://dx.doi.org/10.3349/ymj.2014.55.1.162 | Wrong type publication |
| 242 | [303] | 2013 | González-Pérez J, Sánchez-Leenheer S, Delgado AR, González-Vargas L, Díaz-Zamudio M, Montejo G, et al. Clinical Impact of a 6-Week Preoperative Very Low Calorie Diet on Body Weight and Liver Size in Morbidly Obese Patients. Obes Surg. 2013;23: 1624–1631. doi:10.1007/s11695-013-0977-0 | Without comparison |
| 243 | [304] | 2013 | Dambrauskas Z, Maleckas A, Van Nieuwenhove Y, Thorell A. The effects of short-term preoperative very low calorie diet (VLCD) on long-term outcomes after laparoscopic roux-en-y gastric bypass for morbid obesity. 2013;23: 1184. doi:10.1007/s11695-013-0986-z | Without comparison |
| 244 | [305] | 2013 | Faria SL, Faria OP, A CM, Ito MK, Buffington C. Diet-induced thermogenesis and respiratory quotient after Roux-en-Y gastric bypass surgery: a prospective study. Surg Obes Relat Dis. 2014;10: 138–43. doi:10.1016/j.soard.2013.09.020 | Without comparison |
| 245 | [306] | 2012 | Singhal S, Le DL, Duddempudi S, Anand S. The role of endoscopy in bariatrics: past, present, and future. J Laparoendosc Adv Surg Tech A. 2012;22: 802–11. doi:10.1089/lap.2012.0091 | Wrong intervention |
| 246 | [307] | 2012 | Singh K, Podolsky ER, Um S, Saba S, Saeed I, Aggarwal L, et al. Evaluating the safety and efficacy of BMI-based preoperative administration of low-molecular-weight heparin in morbidly obese patients undergoing Roux-en-Y gastric bypass surgery. Obes Surg. 2012;22: 47–51. | Wrong intervention |
| 247 | [308] | 2012 | Ochner CN, Dambkowski CL, Yeomans BL, Teixeira J, Xavier Pi-Sunyer F. Pre-bariatric surgery weight loss requirements and the effect of preoperative weight loss on postoperative outcome. Int J Obes. 2012;36: 1380–1387. | Wrong outcome |
| 248 | [309] | 2012 | King WC, Hsu JY, Belle SH, Courcoulas AP, Eid GM, Flum DR, et al. Pre- to postoperative changes in physical activity: report from the longitudinal assessment of bariatric surgery-2 (LABS-2). Surg Obes Relat Dis. 2012;8: 522–32. doi:10.1016/j.soard.2011.07.018 | Wrong outcome |
| 249 | [310] | 2012 | Perna M, Romagnuolo J, Morgan K, Byrne TK, Baker M. Preoperative hemoglobin A1c and postoperative glucose control in outcomes after gastric bypass for obesity. Surgery for Obesity and Related Diseases. 2012;8: 685–690. | Wrong outcome |
| 250 | [311] | 2012 | Fullmer MA, Abrams SH, Hrovat K, Mooney L, Scheimann AO, Hillman JB, et al. Nutritional strategy for adolescents undergoing bariatric surgery: report of a working group of the Nutrition Committee of NASPGHAN/NACHRI. J Pediatr Gastroenterol Nutr. 2012;54: 125–35. doi:10.1097/MPG.0b013e318231db79 | Wrong outcome |

| | | | | |
|---|---|---|---|---|
| 251 | [312] | 2012 | Damms-Machado A, Friedrich A, Kramer KM, Stingel K, Meile T, Küper MA, et al. Pre- and postoperative nutritional deficiencies in obese patients undergoing laparoscopic sleeve gastrectomy. Obes Surg. 2012;22: 881–9. doi:10.1007/s11695-012-0609-0 | Wrong outcome |
| 252 | [313] | 2012 | NCT01748682. Very Low Calorie Liquid Diet for Pre op Patients. 2012. Available: https://www.cochranelibrary.com/central/doi/10.1002/central/CN-01539311/full | Protocol |
| 253 | [314] | 2012 | NCT01652105. Randomized Trial of Preoperative Diets Before Bariatric Surgery. 2012. Available: https://www.cochranelibrary.com/central/doi/10.1002/central/CN-01478336/full | Protocol |
| 254 | [315] | 2012 | Ferguson Y, Tham J, Mahon D, Welbourn R, Coulman KD, Knight A. Nurse Specialist and Dietitian-led pre-operative education sessions result in enhanced patient knowledge and preparation prior to bariatric surgery. 2012. | Without comparison |
| 255 | [316] | 2011 | Harbottle L. Audit of nutritional and dietary outcomes of bariatric surgery patients. Obes Rev. 2011;12: 198–204. doi:10.1111/j.1467-789X.2010.00737.x | Wrong outcome |
| 256 | [317] | 2011 | Brody F, Vaziri K, Garey C, Shah R, LeBrun C, Takurukura F, et al. Preoperative liver reduction utilizing a novel nutritional supplement. J Laparoendosc Adv Surg Tech A. 2011;21: 491–5. doi:10.1089/lap.2010.0559 | Wrong outcome |
| 257 | [318] | 2011 | Brody F, Vaziri K, Garey C, Shah R, LeBrun C, Takurukura F, et al. Preoperative Liver Reduction Utilizing a Novel Nutritional Supplement. Journal of Laparoendoscopic & Advanced Surgical Techniques. 2011;21: 491–495. doi:10.1089/lap.2010.0559 | Without comparison |
| 258 | [319] | 2011 | Collins J, McCloskey C, Titchner R, Goodpaster B, Hoffman M, Hauser D, et al. Preoperative weight loss in high-risk superobese bariatric patients: a computed tomography-based analysis. Surgery for Obesity and Related Diseases. 2011;7: 480–485. doi:10.1016/j.soard.2010.09.026 | Without comparison |
| 259 | [320] | 2011 | Kullberg J, Sundbom M, Haenni A, Freden S, Johansson L, Börnert P, et al. Gastric bypass promotes more lipid mobilization than a similar weight loss induced by low-calorie diet. J Obes. 2011;2011: 959601. doi:10.1155/2011/959601 | Without comparison |
| 260 | [321] | 2011 | Faria SL, Faria OP, Buffington C, de Almeida Cardeal M, Ito MK. Dietary protein intake and bariatric surgery patients: a review. Obes Surg. 2011;21: 1798–805. doi:10.1007/s11695-011-0441-y | Without comparison |
| 261 | [322] | 2011 | Harbottle L. Audit of nutritional and dietary outcomes of bariatric surgery patients. Obes Rev. 2011;12: 198–204. doi:10.1111/j.1467-789X.2010.00737.x | Without comparison |
| 262 | [323] | 2011 | Costa Fortes R, Carvalho Garbi Novaes MR. The effects of Agaricus sylvaticus fungi dietary supplementation on the metabolism and blood pressure of patients with colorectal cancer during post surgical phase. Nutr Hosp. 2011;26: 176–86. Available: http://scielo.isciii.es/scielo.php?script=sci_arttext&nrm=iso&lng=pt&tlng=pt&pid=S0212-16112011000100021 | Without comparison |
| 263 | [324] | 2011 | Triffoni-Melo Ade T, Dick-de-Paula I, Portari G V, Jordao AA, Garcia Chiarello P, Diez-Garcia RW. Short-term carbohydrate-restricted diet for weight loss in severely obese women. 2011;21: 1194-1202. doi:10.1007/s11695-010-0110-6 | Without comparison |
| 264 | [325] | 2011 | Woodard GA, Downey J, Hernandez-Boussard T, Morton JM. Impaired alcohol metabolism after gastric bypass surgery: a case-crossover trial. J Am Coll Surg. 2011;212: 209–14. doi:10.1016/j.jamcollsurg.2010.09.020 | Wrong type publication |
| 265 | [326] | 2010 | Schouten R, Rijs CS, Bouvy ND, Hameeteman W, Koek GH, Janssen IM, et al. A multicenter, randomized efficacy study of the EndoBarrier Gastrointestinal Liner for presurgical weight loss prior to bariatric surgery. Ann Surg. 2010;251: 236–43. doi:10.1097/SLA.0b013e3181bdfbff | Wrong intervention |
| 266 | [327] | 2010 | Saruç M, Böler D, Karaarslan M, Baysal Ç, Rasa K, Çakmakçi M, et al. Intragastric balloon treatment of obesity must be combined with bariatric surgery: a pilot study in Turkey. Turk J Gastroenterol. 2010;21: 333–7. doi:10.4318/tjg.2010.0117 | Wrong intervention |
| 267 | [328] | 2010 | Atlas H, Moustarah F, Anvari M, Wiebe S, Graham PJ, Vizhul A, et al. Canadian Surgery Forum1 Laparoscopic sleeve gastrectomy: superobese patients (BMI> 50 kg/m2) may require a second stage procedure2 Weight loss after duodenal switch without gastrectomy for the treatment of severe obesity: review of a single-institution case series of duodeno-ileal intestinal bypa... Canadian Journal of Surgery. 2010;53: S51–S104. | Wrong type publication |
| 268 | [329] | 2009 | Aberle J, Freier A, Busch P, Mommsen N, Beil FU, Dannheim V, et al. Treatment with sibutramine prior to Roux-en-Y gastric bypass leads to an improvement of metabolic | Wrong intervention |

| | | | | |
|---|---|---|---|---|
| | | | parameters and to a reduction of liver size and operative time. Obes Surg. 2009;19: 1504–7. doi:10.1007/s11695-009-9940-5 | |
| 269 | [330] | 2009 | Breznikar B, Dinevski D. Bariatric surgery for morbid obesity: pre-operative assessment, surgical techniques and post-operative monitoring. Journal of International Medical Research. 2009;37: 1632–1645. | Wrong intervention |
| 270 | [331] | 2009 | Alger-Mayer S, Rosati C, Polimeni JM, Malone M. Preoperative binge eating status and gastric bypass surgery: a long-term outcome study. Obes Surg. 2009;19: 139–145. | Wrong outcome |
| 271 | [332] | 2009 | S de K, Hüsler R, Banic A, Constantinescu MA. Body contouring surgery following bariatric surgery and dietetically induced massive weight reduction: a risk analysis. Obes Surg. 2009;19: 553–9. doi:10.1007/s11695-008-9659-8 | Without comparison |
| 272 | [333] | 2008 | Fujioka K, Yan E, Wang H-J, Li Z. Evaluating preoperative weight loss, binge eating disorder, and sexual abuse history on Roux-en-Y gastric bypass outcome. Surgery for Obesity and Related Diseases. 2008;4: 137–143. | Wrong outcome |
| 273 | [334] | 2008 | NCT00623792. Study on Impact of Lifestyle Change and Weight Loss Before Bariatric Surgery. 2008. Available: https://www.cochranelibrary.com/central/doi/10.1002/central/CN-02031284/full | Protocol |
| 274 | [335] | 2008 | Kreft JS, Montebelo J, Fogaça KCP, Rasera I, Oliveira MRM. Gastric bypass: post-operative complications in individuals with and without preoperative dietary guidance. J Eval Clin Pract. 2008;14: 169–171. | Without comparison |
| 275 | [336] | 2008 | King WC, Belle SH, Eid GM, Dakin GF, Inabnet WB, Mitchell JE, et al. Physical activity levels of patients undergoing bariatric surgery in the Longitudinal Assessment of Bariatric Surgery study. Surg Obes Relat Dis. 2008;4: 721–8. doi:10.1016/j.soard.2008.08.022 | Wrong type publication |
| 276 | [337] | 2008 | Panagi Z, Bountouris P, Papadimitriou E, Skouroliakou M, Kalfarentzos F. Preliminary evaluation of preoperative and short-term (1 year) postoperative serum fat-soluble vitamin levels in super-obese patients undergoing A rouxen-Y gastric bypass with biliopancreatic diversion (RYGBP/BPD) malabsortive operation. REVIEW OF CLINICAL PHARMACOLOGY AND PHARMACOKINETICS-INTERNATIONAL EDITION-. 2008;22: 264. | Wrong type publication |
| 277 | [338] | 2007 | Kroh M, Liu R, Chand B. Laparoscopic bariatric surgery: what else are we uncovering? Liver pathology and preoperative indicators of advanced liver disease in morbidly obese patients. Surg Endosc. 2007;21: 1957–1960. | Wrong population |
| 278 | [339] | 2007 | Pires LV, Martins LM, Geloneze B, Tambascia MA, Hadad do Monte SJ, do Nascimento Nogueira N, et al. The effect of Roux-en-Y gastric bypass on zinc nutritional status. Obes Surg. 2007;17: 617–21. Available: https://pesquisa.bvsalud.org/portal/resource/es/mdl-17658020 | Wrong outcome |
| 279 | [340] | 2007 | Benjaminov O, Beglaibter N, Gindy L, Spivak H, Singer P, Wienberg M, et al. The effect of a low-carbohydrate diet on the nonalcoholic fatty liver in morbidly obese patients before bariatric surgery. Surg Endosc. 2007;21: 1423–1427. doi:10.1007/s00464-006-9182-8 | Without comparison |
| 280 | [341] | 2006 | Quadros MRR, Bruscato GT, Branco Filho AJ. Compulsão alimentar em pacientes no pré-operatório de cirurgia bariátrica. Psicol argum. 2006;24: 59–65. Available: https://periodicos.pucpr.br/psicologiaargumento/article/view/20159/19449 | Other language |
| 281 | [342] | 2006 | Jamal MK, DeMaria EJ, Johnson JM, Carmody BJ, Wolfe LG, Kellum JM, et al. Insurance-mandated preoperative dietary counseling does not improve outcome and increases dropout rates in patients considering gastric bypass surgery for morbid obesity. Surgery for obesity and related diseases. 2006;2: 122–127. | Wrong outcome |
| 282 | [343] | 2006 | Lewis M, Phillips M, Slavotinek J, Kow L, Thompson C, Toouli J. Change in Liver Size and Fat Content after Treatment with Optifast<SUP>®</SUP> Very Low Calorie Diet. Obes Surg. 2006;16: 697–701. doi:10.1381/096089206777346682 | Without comparison |
| 283 | [344] | 2006 | Colles SL, Dixon JB, Marks P, Strauss BJ, O’Brien PE. Preoperative weight loss with a very-low-energy diet: quantitation of changes in liver and abdominal fat by serial imaging1–3. Am J Clin Nutr. 2006;84: 304–311. doi:10.1093/ajcn/84.1.304 | Without comparison |
| 284 | [345] | 2006 | Lewis MC, Phillips ML, Slavotinek JP, Kow L, Thompson CH, Toouli J. Change in liver size and fat content after treatment with Optifast very low calorie diet. Obes Surg. 2006;16: 697–701. doi:10.1381/096089206777346682 | Without comparison |

| | | | | |
|---|---|---|---|---|
| 285 | [346] | 2006 | Silver HJ, Torquati A, Jensen GL, Richards WO. Weight, dietary and physical activity behaviors two years after gastric bypass. Obes Surg. 2006;16: 859–64. doi:10.1381/096089206777822296 | Without comparison |
| 286 | [347] | 2005 | Jamal MK, DeMaria EJ, Johnson JM, Carmody BC, Wolfe LG, Kellum JM, et al. Preoperative dietary counseling does not improve outcome and increases drop-out rates in patients considering gastric bypass surgery for morbid obesity. Surgery for Obesity and Related Diseases. 2005;1: 223. | Wrong outcome |
| 287 | [348] | 2005 | Yago MD, González V, Serrano P, Calpena R, Martínez MA, Martínez-Victoria E, et al. Effect of the type of dietary fat on biliary lipid composition and bile lithogenicity in humans with cholesterol gallstone disease. 2005;21: 339-347. doi:10.1016/j.nut.2004.06.028 | Without comparison |
| 288 | [349] | 2005 | Jamal MK, DeMaria EJ, Johnson JM, Carmody BC, Wolfe LG, Kellum JM, et al. Preoperative dietary counseling does not improve outcome and increases drop-out rates in patients considering gastric bypass surgery for morbid obesity. Surgery for Obesity and Related Diseases. 2005;1: 223. | Without comparison |
| 289 | [350] | 2004 | Fris RJ. Preoperative Low Energy Diet Diminishes Liver Size. Obes Surg. 2004;14: 1165–1170. doi:10.1381/0960892042386977 | Without comparison |
| 290 | [351] | 2004 | Solga S, Alkhuraishe AR, Clark JM, Torbenson M, Greenwald A, Diehl AM, et al. Dietary composition and nonalcoholic fatty liver disease. Dig Dis Sci. 2004;49: 1578–83. doi:10.1023/b:ddas.0000043367.69470.b7 | Without comparison |
| 291 | [352] | 2004 | Busetto L, Segato G, M DL, Bortolozzi E, MacCari T, Magon A, et al. Preoperative weight loss by intragastric balloon in super-obese patients treated with laparoscopic gastric banding: a case-control study. Obes Surg. 2004;14: 671–6. doi:10.1381/096089204323093471 | Wrong type publication |
| 292 | [353] | 1998 | Ribeiro RP, De Oliveira LM, Dos Santos JE. Selection of an intact casein or casein hydrolysate diet by rats submitted to protein deprivation and bowel resection. Physiol Behav. 1998;63: 185–9. Available: https://pesquisa.bvsalud.org/portal/resource/es/mdl-9423957 | Wrong type publication |
| 293 | [354] | 1983 | Coughlin K, Bell RM, Bivins BA, Wrobel S, Griffen WO. Preoperative and postoperative assessment of nutrient intakes in patients who have undergone gastric bypass surgery. Archives of Surgery. 1983;118: 813–816. | Wrong population |
| 294 | [355] | 1983 | Coughlin K, Bell RM, Bivins BA, Wrobel S, Griffen WO. Preoperative and postoperative assessment of nutrient intakes in patients who have undergone gastric bypass surgery. Archives of Surgery. 1983;118: 813–816. | Wrong intervention |
| 295 | [356] | 1947 | Harry G V. Gastro-enterostomy from the economic viewpoint; a plea for its more extensive use, with a review of 104 cases. Carib Med J. 1947;9: 21–31. Available: https://pesquisa.bvsalud.org/portal/resource/es/med-3950 | Wrong type publication |

**Table S3: Summary of findings table using the GRADE approach**

**Preoperative nutritional interventions on surgical outcomes on metabolic and bariatric surgery**

**Patient or population:** Patients undergoing metabolic or bariatric surgery

**Intervention:** Low and very low-calorie diets

**Comparison:** Regular diet

| Outcomes | № of participants (studies) Follow-up | Certainty of the evidence (GRADE) | Relative effect (95% CI) | Anticipated absolute effects | |
|---|---|---|---|---|---|
| | | | | Risk with Regular diet | Risk difference with Low and very low-calorie diets |
| Any type of complications | 1197 (4 randomized trials; 4 non-randomized studies) | ⊕◯◯◯ Very low[a,b,c] | **OR 0.78** (0.49 to 1.26) | 100 per 1000 | **20 fewer per 1000** (49 fewer to 23 more) |
| Operative time (minutes) | 748 (2 randomized trials; 3 non-randomized studies) | ⊕◯◯◯ Very low[a,c,d] | - | The median operative time (minutes) was **97.3** minutes | MD **2.75 minutes lower** (4.24 lower to 1.26 lower) |
| Length of hospital stay (days) | 636 (4 non-randomized studies) | ⊕◯◯◯ Very low[a,c,d] | - | The median length of hospital stay (days) was **3.65** days | MD **0.17 days lower** (0.26 lower to 0.09 lower) |

***The risk in the intervention group** (and its 95% confidence interval) is based on the assumed risk in the comparison group and the **relative effect** of the intervention (and its 95% CI).

**CI:** confidence interval; **MD:** mean difference; **OR:** Odds ratio

**GRADE Working Group grades of evidence**
**High certainty:** we are very confident that the true effect lies close to that of the estimate of the effect.
**Moderate certainty:** we are moderately confident in the effect estimate: the true effect is likely to be close to the estimate of the effect, but there is a possibility that it is substantially different.
**Low certainty:** our confidence in the effect estimate is limited: the true effect may be substantially different from the estimate of the effect.
**Very low certainty:** we have very little confidence in the effect estimate: the true effect is likely to be substantially different from the estimate of effect.

a. The certainty of the evidence was downgraded in one level for risk of bias, the blinding of participants or personnel were high in most included trials. b. The certainty of evidence was downgraded one level due to imprecision since each end of the confidence interval leads to a different decision. c. The certainty of evidence was downgraded for imprecision, as the studies together had a small sample size. d. The certainty of evidence was downgraded one level due to inconsistency; the heterogeneity is substantial ($I^2$ >50%).
